# Low density marker‐based effectiveness and efficiency of early‐generation genomic selection relative to phenotype‐based selection in dolichos bean (*Lablab purpureus* L. Sweet)

**DOI:** 10.1002/tpg2.70039

**Published:** 2025-05-26

**Authors:** Mugali Pundalik Kalpana, Sampangi Ramesh, Chindi Basavaraj Siddu, Gonal Basanagouda, K. Madhusudan, Hosakoti Sathish, Dinesh Sindhu, Munegowda Kemparaju, C. Anilkumar

**Affiliations:** ^1^ Department of Genetics and Plant Breeding, College of Agriculture University of Agricultural Sciences Bangalore India; ^2^ Central Sericultural Research and Training Institute Pampore India; ^3^ ICAR‐National Rice Research Institute Cuttack India; ^4^ Department of Agronomy and Plant Genetics University of Minnesota Saint Paul Minnesota USA

## Abstract

Genomic prediction has been demonstrated to be an efficient approach for the selection of candidates based on marker information in many crops. However, efforts to understand the efficiency of genomic selection over phenotype‐based selection in understudied crops such as dolichos bean (*Lablab purpureus* L. Sweet) are limited. Our objectives were to (i) explore the effective marker density for achieving high prediction accuracy and (ii) assess the effectiveness and efficiency of genomic selection over phenotype‐based selection on seed yield at early segregating generations in dolichos bean. In this study, the training population, which consisted of F_5:6_ recombinant inbreds, had a shared common parent with the breeding population, which consisted of F_2_ generation breeding population. The populations were genotyped with newly synthesized genomic simple sequence repeat‐based markers. The effective marker density for genomic prediction was assessed by using a varying number of markers in predictions using 11 different models. Furthermore, the effectiveness of genomic selection was assessed by comparing the genetic gains in progenies between genotypes selected based on predicted seed yield and phenotypically selected genotypes. Our results indicate that low‐density markers that are evenly distributed throughout the genome are sufficient for the integration of genomic selection in dolichos breeding programs. The genomic selection was proved to be two times more effective than phenotypic selection in early‐generation selection in dolichos beans. The results have a significant impact on adopting genomic selection in regular breeding programs of Dolichos beans at a low cost.

## Core Ideas

- Genomic selection in understudied crops like dolichos bean is effective.
- Low marker density is sufficient to achieve relatively high prediction accuracy in dolichos bean.
- Early‐generation selection using genomic prediction is effective for improving genetic gain in seed yield.
- Genomic selection shifts the allele frequency more rapidly than phenotypic selection in early generations.

**Abbreviations**
BLUE: best linear unbiased estimator
BP: breeding population
BRR: Bayesian ridge regression
CV: cross validation
GEBV: genomic estimated breeding value
GS: genomic selection
LASSO: least absolute shrinkage and selection operator
LD: linkage disequilibrium
MAS: marker‐assisted selection
PA: prediction accuracy
PS: phenotypic basedselection
QTL: quantitative trait locus
RFR: random forest regression
rh^2^
: realized heritability
RIL: recombinant inbred line
RKHS: reproducing kernel Hilbert spaces regression
RPA: realized prediction accuracy
rrBLUP: ridge regression best linear unbiased predictor
RS: realized selection response
SD: selection differential
SNP: single nucleotide polymorphism
SPLS: sparse partial least squares
SSR: simple sequence repeats
SVR: support vector regression
TBVs: true breeding values
TP: training population
TS: training set
VS: validation set

## 1 INTRODUCTION

Dolichos bean (*Lablab purpureus* L. Sweet) is one of the important and ancient food legumes extensively grown in southern India and African countries (Ramesh & Byregowda, 2016). It is commonly known as “hyacinth bean,” “field bean,” “Indian bean,” and so on. It is a self‐pollinated crop with 2*n* = 22 chromosomes (She & Jiang, 2015), constituting a small genome size of 367 Mbp (Iwata et al., 2013), which is less than the rice (*Oryza sativa* L.) genome. It is believed that dolichos bean originated in India (Nene, 2006). It is grown both in rainy and post‐rainy seasons. However, the crop expression and yielding potential of dolichos bean is better in post‐rainy season than in rainy season (Ramesh & Byregowda, 2016). It is a multi‐purpose crop, as it is grown for food and fodder use. When grown for food, fresh beans are used for vegetable purposes, and seeds are used in various culinary preparations. Fresh beans, after the removal of the pod cover, are consumed as a vegetable. When grown for seeds, physiologically matured dry pods are harvestable products. The seeds after pod threshing are a consumable economic product. Both fresh beans and seeds are important sources of protein (22%–28%), especially for those who depend on vegetarian diets (Ramesh & Byregowda, 2016). Recent research findings indicate dolichos bean extracts impede infections of viral diseases such as influenza and SARS‐CoV‐2, which have been declared world pandemics by the World Health Organization (Liu et al., 2020). Breeding for improved seed productivity would contribute to food security and better nutrition, along with increased income for farmers (Ramesh & Byregowda, 2016).

Conventional dolichos bean breeding depends on phenotype‐based selection, especially in early segregating generations, for fresh pod and seed yield and its component traits (Spoorthi, Ramesh, Sunitha, Vedashree, et al., 2024). Breeding for seed yield in dolichos bean by phenotype‐based selection (PS) has been challenging and arguably not very effective due to the factors such as crop‐stage‐specific expression, complex inheritance, significant cross‐over genotype‐by‐environment interactions, and low heritability. DNA markers, owing to their crop stage non‐specificity, simple inheritance, and environmental neutrality, have been known to be helpful in breeding for difficult‐to‐select traits (Mohler & Singrün, 2004). The use of DNA markers as surrogates of difficult‐to‐select traits is widely referred to as marker‐assisted selection (MAS) (Bernardo, 2020). Implementation of MAS requires the priori identification of gene/quantitative trait loci (QTLs) controlling the target trait and markers closely linked to them.

However, empirical evidence in several highly researched crops like maize (*Zea mays* L.), tomato (*Solanum lycopersicum* L.), soybean (*Glycine max* (L.) Merr.), wheat (*Triticum aestivum* L.), sorghum (*Sorghum bicolor*. (L.) Moench), rice, and so on suggests that MAS is effective only for selection and/or introgression of traits controlled by large‐effect QTL (Bernardo, 2008). In practice, quantitative traits, especially in breeding populations (BPs), are controlled by a large number of small‐effect QTLs, commonly known as minor genes (Bernardo, 2008). The use of an alternate form of MAS that enables handling a large number of small‐effects QTL is therefore most appropriate. Genomic selection (GS), as first ever attempted by Bernardo (1994) in maize and proposed by Meuwissen et al. (2001), is such an approach. The GS is defined as the selection of genotyped‐only BP individuals based on their genomic estimated breeding values (GEBVs) predicted using marker effects estimated by fitting statistical models calibrated in genotyped and phenotyped training population (TP), preferably related to BP. Unlike MAS, which is a design approach, GS is a predictive approach wherein the best genotypes are predicted and selected based on cumulative favorable allelic effects. The effectiveness of GS hinges on the accuracy of predictions. The accuracy of predictions depends on several factors, such as the size and genetic constitution of TP, the genetic relatedness between TP and BP, the number of markers, their strength of linkage with traits of interest, trait heritability, and statistical models (Anilkumar et al., 2022; Bernardo, 2020; Crossa et al., 2017; Edwards et al., 2019; Li et al., 2018; Merrick et al., 2022; Plavšin et al., 2021).

In major commercial crops, such as maize, wheat, rice, soybean, and so on, GS is routinely used to enhance the rate of genetic gain (Jarquín et al., 2014). Recent reports such as use of multi‐trait GS for end‐use traits in wheat (Gill et al., 2023) and grain yield in rice (Anilkumar et al., 2025), and integration of phenomics and machine learning approaches with GS in wheat (Kaushal et al., 2024), have contributed to enhanced prediction accuracy (PA) and genetic gain. The effectiveness and efficiency of GS in terms of realized genetic gain should be significantly greater than that of phenotype‐based selection to justify the routine implementation of GS in breeding crops. However, most studies are aimed at evaluating the accuracy of predictions based on cross‐validation (CV) approaches. To the best of our knowledge, only a few researchers have directly compared the effectiveness and efficiency of GS relative to PS in a few major crops like wheat (Heffner et al., 2011; Lozada et al., 2019; Rutkoski et al., 2015), maize (Beyene et al., 2019; Butoto et al., 2022; Combs & Bernardo, 2013; Gesteiro et al., 2023), barley (Sallam & Smith, 2016), soybean (Bandillo et al., 2023), and carrot (Corak et al., 2023) for different traits and reported that progenies of plants selected based on GS performed better than those selected based on PS. However, such studies on direct comparison between GS and PS in understudied crops, including dolichos bean, have not yet been attempted.

Most researchers have used high‐density single nucleotide polymorphism (SNP) markers for genomic prediction and selection in different crops such as soybean (Wartha & Lorenz, 2024), maize (Xiang et al., 2024), rice (Fritsche‐Neto et al., 2024), and wheat (García‐Barrios et al., 2024). The availability of genomic resources like SNP marker arrays and chips for genotyping dolichos bean is limited, and the genotyping‐by‐sequencing approach is not cost‐effective for regular genotyping in this crop. Hence, low‐density, effective simple sequence repeat (SSR) markers are most commonly used in marker‐assisted dolichos bean breeding programs (Spoorthi, Ramesh, Sunitha, Anilkumar, et al., 2024). Therefore, we hypothesize that the implementation of GS using SSR marker data would be more effective than phenotype‐based selection in dolichos bean. Our hypothesis was based on the following arguments: (i) Fewer markers cover almost the entire genome, as the size of the dolichos bean genome is small (367 Mbp), and linkage disequilibrium (LD) extends to a large genomic distance driven by self‐pollination (Spoorthi, Ramesh, Sunitha, Vedashree, et al., 2024), leading to low effective recombination (one‐third of an obligate outcrossing species) (Nordborg & Donnelly, 1997); (ii) SSR markers cover large genomic regions per assay (Hamblin et al., 2007); and (iii) being multiallelic, fewer SSR markers are sufficient to capture variability among the individuals of TP and BP (Hamblin et al., 2007). Use of as low as 200 restriction fragment length polymorphism markers in maize resulted in a high PA of 0.80 for grain yield (Bernardo, 1994) and use of 135 SSR markers in oil palm resulted in fairly high PA of 0.43 for oil yield (Kwong et al., 2017). Oil palm and maize genomes are much larger than dolichos bean genome; hence, these studies support our hypothesis of using low‐density SSR markers for GS in dolichos bean.

Under these premises and to test our hypothesis on the effectiveness of GS based on low‐density SSR markers, the major objectives of our study were (i) to test the effect of different statistical models, marker density, and TP size on the accuracy of predictions, and (ii) to compare the effectiveness and efficiency of low‐density SSR marker‐based GS relative to PS in early generation (F_2_) for seed yield.

**Core Ideas**

- Genomic selection in understudied crops like dolichos bean is effective.
- Low marker density is sufficient to achieve relatively high prediction accuracy in dolichos bean.
- Early‐generation selection using genomic prediction is effective for improving genetic gain in seed yield.
- Genomic selection shifts the allele frequency more rapidly than phenotypic selection in early generations.

## 2 MATERIALS AND METHODS

### 2.1 Development of training and breeding populations

We used recombinant inbred lines (RILs) as TP and early generation (F_2:3_) population as BP in the current investigation. The TP consisted of 135 F_5:6_ RILs derived from biparental cross between two elite genotypes, namely, HA 4 (Ramesh & Byregowda, 2016) and HA 5 (Ramesh et al., 2018). HA 4 and HA 5 are high‐yielding pure‐line varieties released for commercial production by the University of Agricultural Sciences (UAS), Bangalore, India (Table 1). The F_1_s from the HA 4 × HA 5 cross were grown during the 2020 post‐rainy season, and these were selfed to obtain the F_2_ population (Gonal, 2022). The F_2_ plants were advanced to the F_6_ generation by the single seed descent (SSD) method. A total of 135 F_2:6_ RILs derived from the SSD method survived and were used in the present study. The BP consisted of F_2:3_ individuals derived from crossing two elite genotypes, namely, HA 10–8 and HA 5 (Ramesh et al., 2018) (Table 1). The F_1_s from HA 10–8 × HA 5 were grown during the 2021 post‐rainy season, and these were selfed to obtain 144 F_2_ individuals. The F_2_ individuals were selfed during the 2022 summer season to obtain the F_3_ population, consisting of 144 progenies. Both TP and BP shared a common parent (HA 5). The experimental material was developed in the experimental plots of the Department of Genetics and Plant Breeding, College of Agriculture (CoA), UAS, Bangalore, India.

**TABLE 1:** Pedigree/source of parents used to derive training and breeding populations.

Parents	Growth habit	Source	Pedigree/source	References
HA 4	Determinate	Karnataka, India	HA 3 × Magadi local	Ramesh and Byregowda (2016)
HA 5	Indeterminate	Karnataka, India	HA 4 × GL 153	Ramesh et al. (2018)
HA 10‐8	Determinate	Karnataka, India	HA 4 × GL 153	Shivakumar et al. (2016)

### 2.2 Genotyping of TP and BP

Young leaves from 21‐day‐old seedlings were collected from plants in training and BPs. The genomic DNA was isolated by the cetyl‐tri‐methyl ammonium bromide method (Doyle, 1991). Initially, the parental polymorphism between parents of TP (HA 4 and HA 5) and between parents of BP (HA 10–8 and HA 5) was examined using 591 genomic SSR markers. These genomic SSR markers were developed from sequence information of the popular variety HA 4, as described in Spoorthi et al. (2024), and the informativeness of these markers was good enough to capture genome‐wide variation (Spoorthi, Ramesh, Sunitha, Anilkumar, et al., 2024). Out of 591, 140 SSR markers between HA 4 and HA 5 parents and 97 SSR markers between HA 10–8 and HA 5 parents were found to be polymorphic. The polymorphic markers were used to genotype training and BPs in the present study. The amplification of marker alleles was carried out through polymerase chain reaction in thermocyclers from Applied Biosystems/Veriti 96 well in a 35‐cycle program, using the following temperatures and times: 94°C for 1 min (initial denaturation), 94°C for 2 min (cyclic denaturation), the specific temperature of each primer, in °C, for 1 min (annealing), 72°C for 3 min (cyclic extension), 72°C for 10 min (final extension), and products stored at 4°C. The final reaction volume was 14 µL of each sample, being 2 µL of DNA (10 ng/µL), 4 µL of Amplicon PCR Master Mix, and 8 µL of nuclease‐free water. The amplicons were separated on a 3.5% agarose gel, stained with ethidium bromide, and visualized through the photo‐documentation system (Bio‐Imaging Systems). The amplicon sizes specific to HA 4 of TP in the defined product size range were scored as “0” and those specific to HA 5 of TP were scored as “2.” The amplicon sizes specific to HA 10–8 of BP in the defined product size range were scored as “0,” those specific to HA 5 of BP were scored as “2,” and heterozygous genotypes as “1.”

### 2.3 Phenotyping of TP and BP

The TP was evaluated at five locations. The seeds of TP were planted in an alpha lattice design with two replications during the 2022 growing season. At the CoA, Bangalore, India, the trials were conducted in both the rainy and post‐rainy seasons, while at the other four locations—(i) Krishi Vigyana Kendra (KVK), Hadonahalli, Bangalore, India; (ii) CoA, Chintamani, India; (iii) CoA, Hassan, India; and (iv) a farmer's field, Belagavi, Karnataka, India—experiments were conducted only during the post‐rainy season. The seeds of the F_2:3_ families of BP were planted during the rainy and post‐rainy seasons of 2022 in experimental plots in CoA, Bangalore, India. The seeds of each RIL and F_3_ family were planted in a single row of 3 m length and 0.60 m apart. Fifteen days after sowing, seedlings were thinned to maintain 0.2 m between plants within a row. To estimate seed yield per plant, physiologically matured dry pods (when pods turned to tan color) were harvested from 10 randomly selected plants (avoiding border ones) from each RIL family, F_2:3_ family, and individual F_2_ plants. The pods were sun‐dried, hand‐threshed, and weighed. The data on seed yield per plant were recorded on 10 plants in each RIL and F_2:3_ family, avoiding border plants, using standard protocol (Byregowda et al., 2015). The per plant seed yield of corresponding F_2_ plants of BP was recorded and used for phenotype‐based selection. The data on seed yield per plant were used for genomic prediction and selection.

### 2.4 Statistical analysis

#### 2.4.1 Estimation of genetic relatedness between TP and BP

One of the factors that contribute to effectiveness of GS is the genetic relatedness between individuals of TP and BP. The genetic relatedness was quantified using molecular markers based on Wright's fixation index (*F*_st_) (Weir & Cockerham, 1984) and dissimilarity index (Nei, 1978). The analysis was implemented using GenAlEx 6.5 software (Peakall & Smouse, 2006). The *F*_st_ ranges from 0 to 1. A value of “0” indicates no differentiation between populations. In other words, *F*_st_ = 0 indicates a very high magnitude of relatedness between individuals of the TP and the BP. A value of “1” indicates complete differentiation or a high degree of genetic unrelatedness between the individuals of TP and BP (Soumya et al., 2021). Apart from genetic relatedness between individuals of the TP and BP, genetic diversity within TP is also important. This is because, when the TP with narrow genetic diversity was used low prediction accuracies were reported. It is not possible to accurately estimate all the genotypic (marker) effects that explain the variation in the phenotype (Berro et al., 2019; Norman et al., 2018).

#### 2.4.2 Estimation of marker effects

Estimation of marker effects requires phenotypic and genotypic data. The phenotype of individuals of TP evaluated at five locations for seed yield was estimated in terms of best linear unbiased estimators (BLUEs) by considering days to 50% flowering as a covariate to rule out its possible confounding effects on seed yield using a linear mixed model implemented through Meta‐R software (Alvarado et al., 2020). The BLUE estimates of individuals in TP for seed yield were considered phenotypic data, and the SSR marker data of TP were considered genotypic data for estimating marker effects. The components of variance in TP, namely, genotypic (*σ*_g_^2^) and residual variance (*σ*_e_^2^), were estimated using the restricted maximum likelihood approach implemented through Meta‐R software (Alvarado et al., 2020).

The marker effects were estimated using 11 statistical models, which includes two non‐Bayesian models, namely, (i) ridge regression BLUP (rrBLUP) (Meuwissen et al., 2001) and (ii) least absolute shrinkage and selection operator (LASSO) (Tibshirani, 1996), five Bayesian models, namely, (i) Bayes A (Meuwissen et al., 2001), (ii) Bayes B (Spiegelhalter et al., 2002), (iii) Bayes C (Spiegelhalter et al., 2002), (iv) Bayesian LASSO (Yi & Xu, 2008), and (v) Bayesian ridge regression (BRR) (Shi et al., 2016), two semi‐parametric models, namely, (i) sparse partial least squares (SPLS) (Chun & Keleş, 2010) and (ii) reproducing kernel Hilbert spaces regression (RKHS) (Campos et al., 2010), and two machine learning models, namely, (i) support vector regression (SVR) (Cortes & Vapnik, 1995) and (ii) random forest regression (RFR) (Segal, 2004). The assumptions and key features of these models are detailed in Table S1. Except for the rrBLUP model, all other models used in the present study are considered complex models.

The marker effects estimated from the models were used to predict the performance of individuals in BPs. The effectiveness of the selection of individuals in BP based on their predicted performance depends on the accuracy of the prediction. PA was measured as the correlation (denoted as [*r*_gg_]) between true genotypic value and genotypic value predicted from marker effects divided by the square root of trait heritability. CV is the most widely used method of measuring PA. Of the several methods of CV, we used the *K*‐fold method to assess the PA.

#### 2.4.3 *K*‐fold cross‐validation

A fivefold CV scheme was used where the TP was divided into five subsets and individuals from the TP were assigned to each subset randomly. One of the five subsets was designated as a validation set (VS), while the remaining four subsets were designated as training sets (TSs). The phenotype and marker information of four sets was used to predict the seed yield of VS. The analysis was repeated to consider all subsets as VS at least once. This process was repeated with 25,000 iterations to allow the models to converge. The CV process helps evaluate the performance of the model in the TP.

The accuracy of predicted performance is normally quantified as the correlation (*r*_ĝg_) between GEBVs (ĝ) and true breeding values (TBVs) of individuals of VS. However, direct computation of PA for empirical datasets is not possible because TBVs are not known. Hence, the correlation between predicted phenotypic values and observed phenotype values, referred to as predictive ability, is often computed. To indirectly estimate PA, predictive ability is divided by the square root of trait heritability (*h*) (Dekkers, 2007). In the present study, PA was computed as predictive ability/*h*, where “*h*” is the square root of the heritability of seed yield estimated in TP. A higher estimate of correlation was interpreted as greater accuracy of predicted performance.

#### 2.4.4 Effect of marker density and population size on the prediction accuracy

**FIGURE 1:**
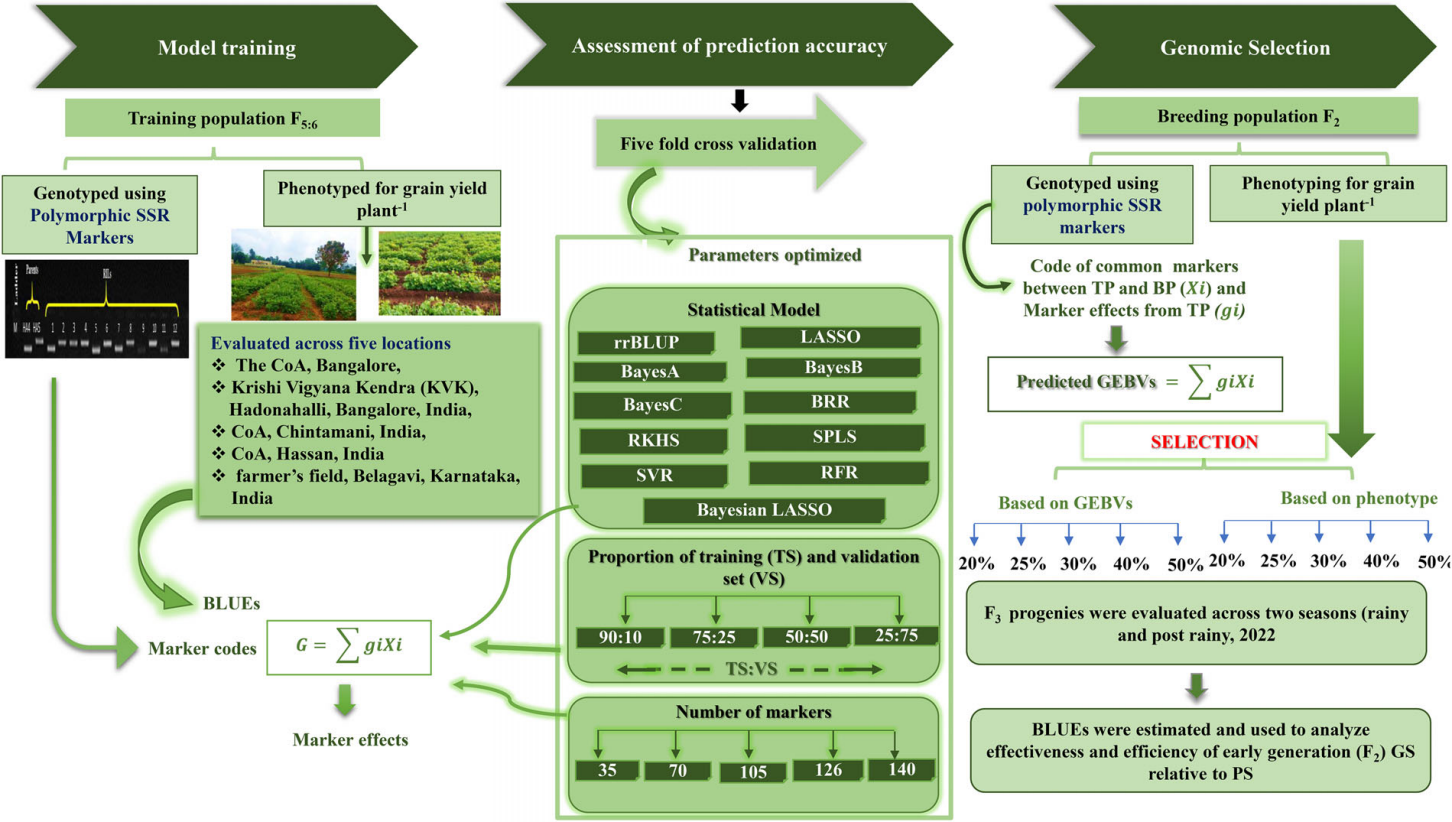
Study design and schematic overview of the methodology followed to test the effectiveness and efficiency of genomic selection (GS) relative to phenotype‐based selection (PS) with low‐density simple sequence repeat (SSR) markers in dolichos bean. BLUEs, best linear unbiased estimator; BP, breeding population; BRR, Bayesian ridge regression; GEBV, genomic estimated breeding value; LASSO, least absolute shrinkage and selection operator; PS, phenotypic selection; RFR, random forest regression; RKHS, reproducing kernel Hilbert spaces regression; rrBLUP, ridge regression best linear unbiased predictor; SPLS, sparse partial least squares; SVR, support vector regression; TP, training population.

The effect of marker density on the accuracy of predicted performance was assessed through the fivefold CV approach. Phenotypic data and a series of randomly selected marker subsets (25%, 50%, 75%, and 90% markers) and the full set of 100% polymorphic SSR marker data from the TP were used to estimate the effect of marker density on the PA. The accuracy of the predicted performance of individuals in TP for each marker subset and the full marker set was estimated using the aforementioned procedure. The same procedure was repeated for different size proportions of TS and VS (25:75, 50:50, 75:25, and 90:10) and for all 11 models under study to assess the effect of TS and VS sizes and statistical model on PA. The flowchart of the entire study design explained so far is presented in Figure 1.

#### 2.4.5 Assessment of effectiveness and efficiency of GS relative to PS

##### Effectiveness of GS relative to PS

To examine the effectiveness of any method of selection like GS, it should be compared with conventional phenotype‐based selection. In our study, we compared GS with PS using two approaches. In the first approach, we compared the mean seed yield per plant of progenies derived from F_2_ plants selected based on predicted performance with those selected based on phenotype. In the second approach, we estimated and compared the shifts in marker allele frequencies in response to GS and PS relative to the unselected F_2_ population.

##### Effectiveness based on mean seed yield of progenies of GS and PS

We selected individuals of F_2_ BP based on their predicted performance and also based on their observed phenotype for seed yield per plant at different intensities (20%, 25%, 30%, 40%, and 50%). The progenies (F_3_) derived from the F_2_ plants selected based on their predicted phenotype and those selected based on their observed phenotype were compared for seed yield. The significance of differences in seed yield between the progenies derived from individuals selected based on GS and PS at different selection intensities was examined by using the following procedure.

The mean seed yield of F_3_ progenies of individuals selected based on GS and PS was estimated as the BLUEs across two seasons by considering days to 50% flowering as a covariate to rule out its possible confounding effects. The analysis was implemented using meta‐R software (Alvarado et al., 2020). The BLUE values of F_2:3_ progenies derived from F_2_ plants selected based on GS and PS were considered for comparing the effectiveness of GS relative to PS. At each of the selection intensities, the significance of differences in mean seed yield of F_3_ progenies resulting from GS and PS was examined using two‐sample “*t*” test with unequal variance (Snedecor & Cochran, 1967).

$$t=\frac{\bar{x}1-\bar{x}2}{\mathrm{Sp}\;\sqrt{\frac{1}{n1}+\frac{1}{n2}}}\;\mathrm{Sp}=\sqrt{\frac{\left(n1-1\right)s_{1}^{2}+\left(n2-1\right)s_{2}^{2}}{n1+n2-2}}$$

where $\bar{x}1$ is the mean seed yield of F_3_ progenies derived from F_2_ plants selected based on predicted phenotype, $\bar{x}2$ is the mean seed yield of F_3_ progenies derived from F_2_ plants selected based on phenotype, Sp is the pooled sample variance, *s*_1_ is the sample variance within F_3_ progenies derived from F_2_ plants selected based on predicted phenotype, *s*_2_ is the sample variance within F_3_ progenies derived from F_2_ plants selected based on phenotype, *n*_1_ is the number of plants selected based on predicted phenotype, and *n*_2_ is the number of plants selected based on predicted phenotype.

##### Effectiveness based on shifts in marker allele frequency

We examined whether the frequency of alleles at SSR marker loci shifted when selection (GS and PS) was applied in the F_2_ population. We first estimated the frequencies of alleles at all marker loci in the F_2_ population. We then estimated the frequencies of alleles at these marker loci in plants selected based on predicted phenotypes and observed phenotypes. The significance of the differences in allele frequencies between the individuals selected based on GS and PS and those of the F_2_ BP was tested using a two‐tailed two‐sample “*t*” test. The following formula was used with the null hypothesis that there is no shift in SSR marker allele frequencies in response to GS and PS relative to that in F_2_ BP (Lebowitz et al., 1987).

$$t=\frac{Fm\left(\mathrm{BP}\right)-Fm\left(\mathrm{SP}\right)}{\sqrt{\frac{p\left(1-p\right)}{2N1}+\frac{p\left(1-p\right)}{2N2}}}$$

where Fm(BP) is SSR marker allele frequency in F_2_ BP, Fm(SP) is SSR marker allele frequency of selected based on predicted and observed phenotype ,p is expected allele frequency (*p* = 0.5), N1 is the number of individuals in F_2_ BP, N2 is the number of F_2_ individuals in selected based on predicted and observed phenotype.

Further, mean allele frequency across all marker loci between GS and PS relative to the F_2_ population was compared using Tukey's honestly significant difference test at *α* = 0.05. The distribution of marker allelic frequency in response to GS and PS relative to F_2_ BP was plotted using line graphs using Microsoft Excel software. A significant shift in the frequencies of alleles at a greater number of marker loci and significant differences in mean allelic frequency in response to GS than those in response to PS would indicate a greater effectiveness of GS than PS.

##### Efficiency of GS relative to PS

The efficiency of GS relative to PS was quantified in terms of realized selection differential (SD), realized response to selection (RS), and realized heritability (rh^2^), realized prediction accuracy (RPA) using the following formulae (Falconer, 1996):

$$\mathrm{Realized}\;\mathrm{response}\;\mathrm{to}\;\mathrm{selection}\;\left(\mathrm{RS}\right)=m_{F_{3}}-m_{F_{2}}$$

where $m_{F_{3}}$ is mean seed yield of F_3_ progenies derived from F_2_ individuals selected based on predicted and observed phenotype, $m_{F_{2}}$ is mean of predicted seed yield (for GS) or observed seed yield (for PS) of F_2_ population.

$$\mathrm{Selection}\;\mathrm{differential}\;\left(\mathrm{SD}\right)=m_{s}-m_{F_{2}}$$

where $m_{s}$ is mean seed yield of F_2_ plants selected based on predicted and observed phenotype, $m_{F_{2}}\;$ is mean of predicted seed yield (for GS) or observed seed yield (for PS) of F_2_ population.

$$\mathrm{Realized}\;\mathrm{heritability}\;\left(rh^{2}\right)=\frac{\mathrm{RS}}{\mathrm{SD}}$$

##### Realized prediction accuracy and phenotypic accuracy

For GS, the RPA was estimated as correlation between predicted GEBVs and observed phenotype of F_2_ individuals divided by square root of heritability. For PS, the realized phenotypic accuracy was estimated as square root of seed yield heritability in F_2_ population. Greater SD, RS, rh^2^, and RPA estimates would indicate a greater efficiency of GS over PS. Relative efficiency of GS versus PS was quantified using the following formula:

$$\mathrm{Relative}\;\mathrm{efficiency}=\frac{\mathrm{RS}\;\mathrm{to}\;\mathrm{GS}}{\mathrm{RS}\;\mathrm{to}\;\mathrm{PS}}$$

Greater this ratio would indicate a greater efficiency of GS relative to PS.

## 3 RESULTS

The pooled analysis of variance (ANOVA) of F_2:6_ RIL as the TP (Table 2) and F_2:3_ families as the BP (Table 3) indicated significant differences among RIL (*p* value = 1.20 × 10^−212^) and those among F_2:3_ families (*p* value = 4.20 × 10^−17^). The RILs showed differential performance across spatial environments (locations), as indicated by the significant mean squares attributable to genotypes × location interactions (*p* value = 9.11 × 10^−16^). However, the performance of F_2:3_ was comparable across seasons, as indicated by the nonsignificant mean squares due to genotypes × seasons interaction (*p* value = 1.00). The performance of RILs was affected by the presence of random error as evidenced by the significant variation due to replications (*p* value = 1.59 × 10^−29^) and blocks (*p* value = 3.40 × 10^−2^). However, the performance of F_2:3_ was not affected by the presence of random error as evidenced by the nonsignificant variation due to replications (*p* value = 0.16) and blocks (*p* value = 0.75). The genetic variation among F_2_ was greater than that among RILs, as evident from the estimates of range and genotypic variance (Table 4). The lower *F*_st_ value and higher Nei's similarity coefficient indicated a higher genetic relationship between individuals of the TP and the BP (Table 5).

**TABLE 2:** Pooled analysis of variance of recombinant inbred lines (training population [TP]) derived from HA 4 × HA 5 for grain yield per plant.

	Degrees of freedom	Mean sum of squares	“*F*” statistic	“*F*” probability
Spatial environment (locations)	04	3144.2	249.31	5.12 × 10^−126^
Replication (location)	05	412.40	32.70	1.59 × 10^−29^
Blocks	70	17.10	1.36	3.40 × 10^−2^
Genotypes	134	436.10	34.58	1.20 × 10^−212^
Genotypes × locations	536	24.7	1.96	9.11 × 10^−16^
Residual	600	12.60		

**TABLE 3:** Pooled analysis of variance of F_3_ progenies (breeding population [BP]) derived from HA 10‐8 × HA 5 for grain yield per plant.

	Degrees of freedom	Mean sum of squares	“*F*” statistic	“*F*” probability
Temporal environment (seasons)	01	1620.91	42.58	3.23 × 10^−10^
Replication (seasons)	02	70.26	01.85	0.16
Blocks	10	25.49	00.67	0.75
Genotypes	143	122.70	03.22	4.20 × 10^−17^
Genotypes × seasons	143	10.17	00.26	1.00
Residual	276	38.06		

**TABLE 4:** Descriptive statistics for grain yield per plant in training and breeding populations.

Population	Generation	Range		Genotypic variance	Genotype × location variance	Residual variance
		Min	Max			
Training population	F_5:6_	14.33	49.73	41.12	5.80	12.60
Breeding population	F_2_	8.53	42.35	73.34	–	28.17

**TABLE 5:** Estimates of Wright's *F*st fixation index and Nei's genetic similarity coefficients as measures of genetic relatedness between training population (TP) and breeding population (BP).

	*F*_st_ value	Nei's
Genetic relatedness between TP and BP	0.043	0.90

### 3.1 Effect of TS and VS size on the prediction accuracy

**FIGURE 2:**
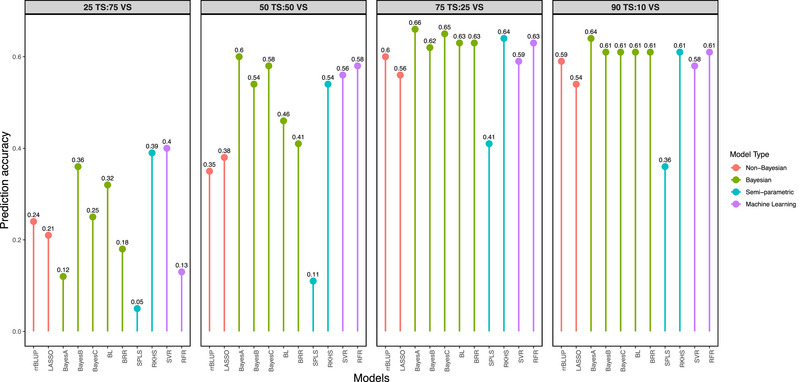
Effect of training set (TS) and validation set (VS) size on the accuracy of predictions. BL, Bayesian LASSO, BRR, Bayesian ridge regression; LASSO, least absolute shrinkage and selection operator; RFR, random forest regression; RKHS, reproducing kernel Hilbert spaces regression; rrBLUP, ridge regression BLUP; SPLS, sparse partial least squares; SVR, support vector regression.

The proportion of TS:VS within the TP was progressively reduced by dividing the TP into 90:10, 75:25, 50:50, and 25:75 in favor of TS and VS, respectively. The estimates of PA were highest at 75 TS:25 VS (ranged from 0.41 to 0.66 across models). The accuracy of predictions increased as the size of TS increased from 34 to 100, and thereafter no further significant increase was observed (Figure 2). The estimates of PA based on Bayes A (0.66) were higher than those predicted based on other models across all the TS:VS proportions. In the 75 TS:25 VS proportion of TP, all the models performed better except SPLS (0.41). However, the performance of rrBLUP (0.60) was comparable to that of other complex models. At a lower TS:VS (50:50) proportion, the complex models performed relatively better compared to the less complex models (Figure 2).

### 3.2 Effect of marker density on the prediction accuracy

**FIGURE 3:**
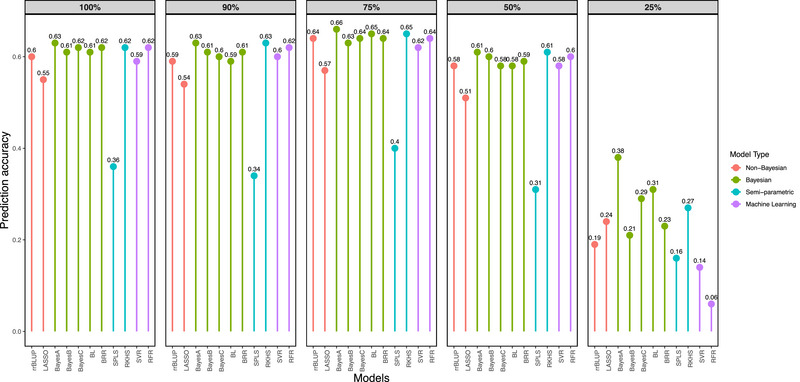
Effect of marker density on the accuracy of predictions. BL, Bayesian LASSO, BRR, Bayesian ridge regression; LASSO, least absolute shrinkage and selection operator; RFR, random forest regression; RKHS, reproducing kernel Hilbert spaces regression; rrBLUP, ridge regression BLUP; SPLS, sparse partial least squares; SVR, support vector regression.

The PA of all the models at marker densities ranging from 100% (a full set of 140 markers) to 25% (35 markers) was estimated using the CV approach. The PA of all the models was highest at a density of 75% (ranged from 0.40 to 0.66 across models) markers across the dolichos genome, and there was no further significant increment in PA upon increasing marker number to 100% (ranged from 0.36 to 0.63 across models) (Figure 3). The accuracy of predictions based on Bayes A (0.63) was higher than that of other models across all the marker densities. At 75% marker density, all the models performed better except SPLS (0.40). As is true with the 50 TS:50 VS proportion, the performance of rrBLUP (0.64) was comparable to that of other complex models.

### 3.3 Effect of statistical model on the prediction accuracy

At optimum marker density (75%) and at optimum TS:VS proportion (75 TS:25 VS), the estimates of accuracy of GEBVs predicted based on Bayesian models, namely, Bayes A (0.66, 0.66), Bayes B (0.63, 0.62), Bayes C (0.64, 0.65), Bayesian LASSO (0.65, 0.63), and BRR (0.64, 0.63), were greater than those predicted based on other models in dolichos bean. The PA was highest for the Bayes A model (0.66) (Figures 2 and 3). Among semi‐parametric models, the RKHS model (0.65, 0.64) outperformed the SPLS model (0.31, 0.41). Among machine learning models, the RFR model (0.64, 0.63) marginally outperformed the SVR model (0.62, 0.59). The estimates of PA based on rrBLUP (0.64, 0.60) were comparable even to complex models such as Bayesian, RKHS, RFR, and SVR (Figures 2 and 3). The complex models performed better under a lower proportion of TS:VS (with 50 TS:50 VS) and marker density (with 50%).

### 3.4 Effectiveness and efficiency of GS relative to PS

#### 3.4.1 Effectiveness based on performance of GS and PS progenies

**FIGURE 4:**
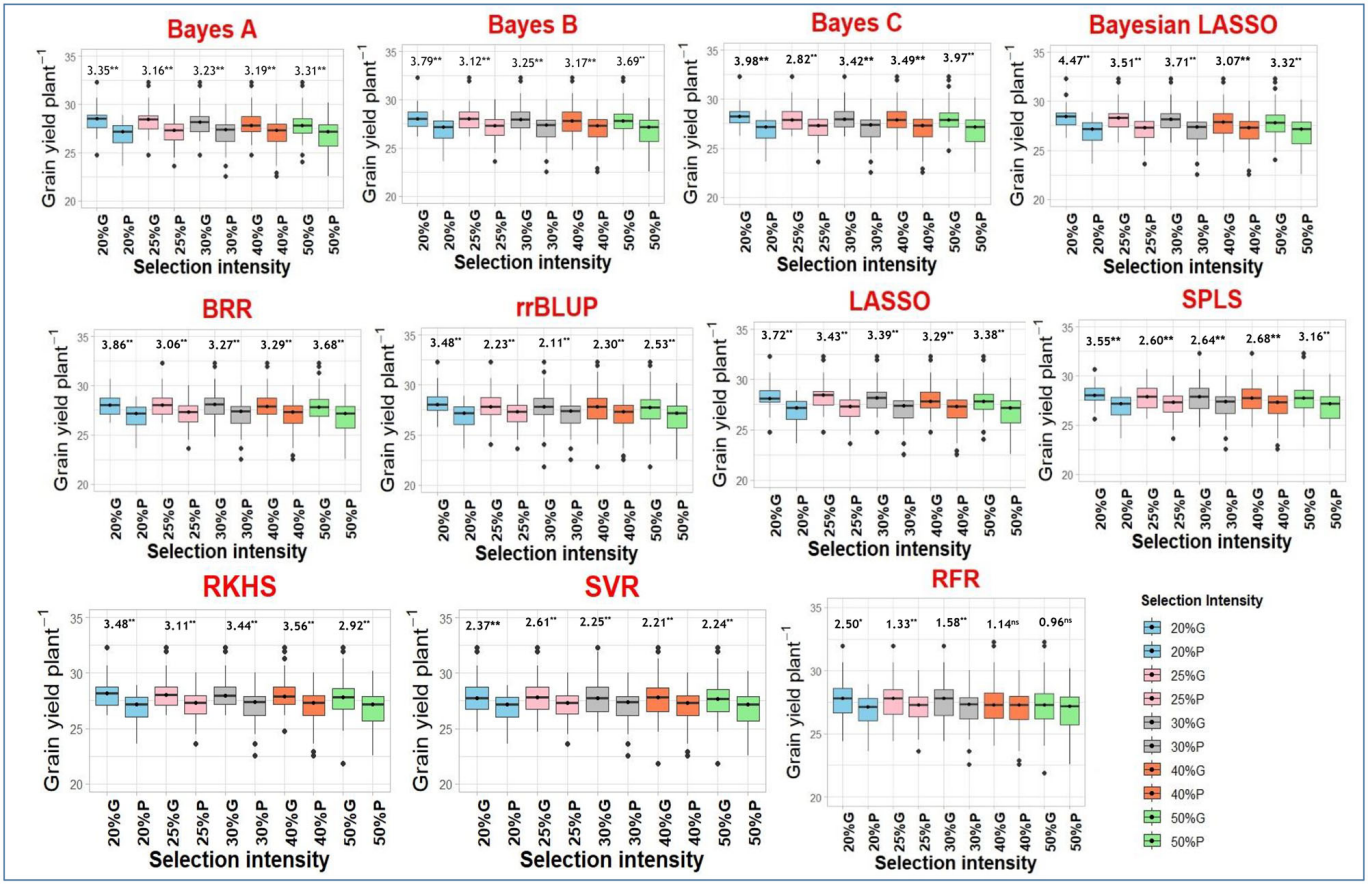
The mean seed yield differences between progenies derived from plants selected based on genomic selection (G) using different models and phenotype‐based selection (P) at different selection intensities. Values indicated on each pair of boxes indicate the *t* statistic values. BL, Bayesian LASSO, BRR, Bayesian ridge regression; LASSO, least absolute shrinkage and selection operator; RFR, random forest regression; RKHS, reproducing kernel Hilbert spaces regression; rrBLUP, ridge regression BLUP; SPLS, sparse partial least squares; SVR, support vector regression. *Significance at *p* = 0.05 and **significance at *p* = 0.01.

The mean seed yield of F_3_ progeny families derived from F_2_ plants selected based on predicted seed yield was significantly greater than those selected based on phenotype as evident from the significant two‐sample “*t*” test at all selection intensities (Figure 4). For instance, the difference in mean seed yield in candidates selected based on the Bayes A model and phenotype‐based selection was 2.21 g at 20% selection intensity, and the difference was significant at *p *= 0.01. The performance of progenies from selection candidates in all the models was significantly (*p *= 0.01) different except for the RFR model, where performance in GS and PS was comparable and nonsignificant at higher selection intensities (at 40% and 50% selection intensities).

#### 3.4.2 Effectiveness based on shift in frequency of alleles at each marker loci

We witnessed significant differences in marker allele frequencies between F_2_ BP and individuals selected based on GS and PS based on two‐sample “*t*” tests. However, these differences in frequency of alleles were observed at a greater number of marker loci (at least at 10 loci) across all the models in response to GS than in response to PS (only at two loci). The number of markers (26) with significant differences in allele frequencies in individuals selected based on marker effects estimated using the rrBLUP model was the highest. For Bayesian models, the number of markers showing significant allelic frequency differences was comparable with the F_2_ population. Among machine learning models, the number of markers with significant allelic differences compared to the F_2_ population was higher for the RFR model (23 markers) than for the SVR model (16 markers). Among semi‐parametric models, the number of markers with significant allelic differences compared to the F_2_ population was higher for the RKHS model (18 markers) than for the SPLS model (10 markers) (Table S2).

#### 3.4.3 Effectiveness based on shift in mean frequency of alleles at marker loci

**FIGURE 5:**
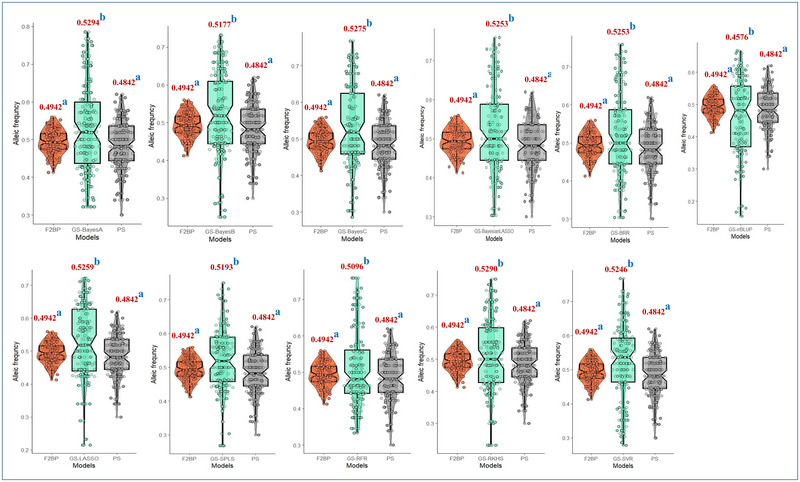
Mean allele frequency difference in response to genomic selection (GS) with different models and phenotypic‐based selection (PS) relative to F_2_ breeding population (BP) based on Tukey's honest significant difference test. Values indicated on top of each box indicate mean allelic frequency. Lowercase letters on plots indicate significance level at least significant difference at 0.05. BL, Bayesian LASSO, BRR, Bayesian ridge regression; LASSO, least absolute shrinkage and selection operator; RFR, random forest regression; RKHS, reproducing kernel Hilbert spaces regression; rrBLUP, ridge regression BLUP; SPLS, sparse partial least squares; SVR, support vector regression.

The selection changed the allele composition of BPs. GS, irrespective of models, accumulated more positive alleles compared to phenotype‐based selection and the F2 population (Figure 5). However, the mean marker allelic frequency was comparable between PS and F_2_ BP. Furthermore, the mean SSR marker allele frequency between PS and GS based on all models differed significantly. All models showed an upward shift in allele frequency except the rrBLUP. The distribution of marker allele frequencies in PS and GS differed from the F_2_ population as well (Figure S1a,b,c). Shifts in marker allele frequency were greater in GS individuals than those in PS individuals.

#### 3.4.4 Efficiency

The estimates of SD, RS, realized heritability, realized prediction, and phenotypic accuracy based on GS were greater than those based on PS, indicating a greater efficiency of GS relative to PS. Further, the efficiency of GS based on rrBLUP, Bayes A, RKHS, and RFR models was almost two times greater than that of PS (Table 6). Realized response to selection was highest in THE Bayes A model (0.67) and lowest in SPLS (0.30). However, realized responses were better in all models compared to selection response in PS.

**TABLE 6:** Estimates of realized selection differential (SD), realized response to selection (RS), realized heritability (rh^2^), and efficiency genomic selection (GS) relative to phenotype‐based selection.

Models	Realized selection differential		Realized response to selection		Realized heritability		Realized prediction and phenotypic accuracy		Relative efficiency of GS relative to PS
	PS	GS	PS	GS	PS	GS	PS	GS	
rrBLUP	0.59	1.13	0.24	0.48	0.39	0.42	0.64	0.75	2.02
Bayes A	0.59	1.32	0.24	0.67	0.39	0.51	0.64	0.74	2.86
Bayes B	0.59	0.94	0.24	0.43	0.39	0.46	0.64	0.77	1.83
Bayes C	0.59	3.14	0.24	0.47	0.39	0.50	0.64	0.72	1.68
BL	0.59	0.71	0.24	0.33	0.39	0.47	0.64	0.67	1.41
BRR	0.59	0.94	0.24	0.45	0.39	0.48	0.64	0.65	1.93
LASSO	0.59	0.98	0.24	0.41	0.39	0.42	0.64	0.66	1.75
SPLS	0.59	0.74	0.24	0.30	0.39	0.41	0.64	0.58	1.28
RKHS	0.59	1.10	0.24	0.52	0.39	0.47	0.64	0.73	2.20
SVR	0.59	0.95	0.24	0.45	0.39	0.47	0.64	0.69	1.91
RFR	0.59	1.08	0.24	0.48	0.39	0.44	0.64	0.76	2.05

Abbreviations: BL, Bayesian LASSO; BRR, Bayesian ridge regression; LASSO, least absolute shrinkage and selection operator; PS, phenotypic selection; RFR, random forest regression; RKHS, reproducing kernel Hilbert spaces regression; rrBLUP, ridge regression best linear unbiased predictor; SPLS, sparse partial least squares; SVR, support vector regression.

## 4 DISCUSSION

In the present investigation, we developed new molecular markers for dolichos bean and explored their effectiveness in GS. We explored their effectiveness in GS based on the optimized model, TP size, TS:VS ratio (75 TS:25 VS), and number of SSR markers (105), and found that our approach was effective at identifying the best genotypes in the F_2_ population. From the field evaluation of populations, the significant differences among RIL and those among F_2:3_ progenies, as indicated by the pooled ANOVA, justify the use of material for the present study (Tables 2 and 3). The variance due to RIL × location interaction suggests differential performance of RILs across locations. These results further suggest the feasibility of identifying trait‐associated markers, thereby paving the way for implementing GS for the genetic improvement of target traits (Kristensen et al., 2018). The use of BLUE values for further analysis helped reduce experimental error and ensure the precision of phenotype data used for model training.

GS is a predictive approach wherein genome‐wide markers are used to predict the phenotype of untested individuals and select those with higher performance without evaluating the trait. The effectiveness and efficiency of GS depend on the accuracy of prediction, which in turn depends on TP composition, its size, and its genetic relatedness to BP (Wang et al., 2012). The accuracy of the predicted phenotype also depends on the statistical model used for prediction, the size of the TS used to estimate marker effects, the density of markers, and the heritability of target traits (Bernardo, 2008; Bernardo & Yu, 2007; Massman et al., 2013; Song et al., 2016). In the present study, the results on the effect of size of the TS and VS, marker density, and different statistical models on PA were investigated, and the effectiveness and efficiency of GS relative to PS were discussed.

### 4.1 Effect of TP size per se on the accuracy of predicted GEBVs

A general consensus does not exist in the literature regarding the size of TP to achieve high PA. However, considering that acceptable PA was achieved in maize biparental populations using as few as 60 individuals (Schaeffer, 2006) and 84 individuals (Riedelsheimer et al., 2013), the use of 135 RIL populations of dolichos bean in the present study for predicting and cross‐validating seed yield per plant is justifiable. Our justification could be further strengthened by theoretical investigation, which suggests that there can be no threshold size (N) of TP below which genomic prediction is ineffective and above which it is effective. Instead, a larger N increases PA, but diminishing returns occur as N becomes too large (Bernardo, 2020). However, as reported by several researchers in different crops (Bassi et al., 2016; Bernardo, 1994), if TP and BP are highly related, a small TP size is sufficient for achieving relatively high PA. In this context, in our study, as TP and BP are highly related, as evidenced by the low estimate of *F*_st_ and the higher estimate of Nei's genetic similarity, high PA was achieved even with as few as 100 individuals in TP. Similar results have also been reported by several previous researchers on different crops. To quote a few, for seedling dry weight in Arabidopsis, the increase in PA ceased when N increased from 48 to 96 (0.40 to 0.73), but was not as steep when the starting value of N was larger (Lorenzana & Bernardo, 2009).

### 4.2 Effect of TS:VS size on prediction accuracy

In our study, PA progressively decreased as the TS:VS proportion was reduced. The highest PA was achieved with a TS:VS proportion of 75:25, suggesting that while larger TSs enhance the accuracy of GEBV predictions, there is an optimal TS:VS ratio that maximizes PA (Figure 2). Among the 11 models evaluated, the Bayes A model consistently performed well in terms of PA. However, the PA based on the rrBLUP model was comparable to that of the more complex Bayes A model, indicating its effectiveness. These results suggest that implementing GS based on cross‐validated TP using the rrBLUP approach at a 75 TS:25 VS proportion appears to be more appropriate in dolichos bean.

### 4.3 Effect of marker density on the prediction accuracy

A rather greater magnitude of PA with a low density of SSR markers in dolichos bean is comparable to that reported in other crops. The PA for seed yield per plant in dolichos bean was high even with as few as 105 SSR markers (Figure 3). The PA did not significantly change beyond 105 markers. The saturation of marker density at 105 markers in dolichos bean could be attributed to small genome size (Iwata et al., 2013) and high LD driven by lower effective recombination due to self‐pollination. Lorenzana and Bernardo (2009) in the barley doubled haploid population demonstrated that the PA increased with the increase in the number of markers, but the increase was greater only at low marker densities. For example, in wheat, reducing the number of SNP markers from 1158 to 192 resulted in only 10% loss in PA (Heffner et al., 2011). Similarly, in six‐row barley breeding germplasm, reducing the number of SNP markers from 73147 to 7142 and from 1023 to 384, respectively, did not significantly impact PA on average (Lorenz et al., 2012). In soybean, Ma et al. (2016) reported a PA of 0.47 for seed yield with a haplotype block rather than with random or equidistant marker sampling. Similarly, Duhnen et al. (2017) reported a soybean PA of 0.39 for seed yield with a moderate‐to‐high density of SNP markers. Roorkiwal et al. (2016) reported a PA of 0.14 for seed yield in chickpeas. In rice, for several traits, there were hardly any differences in PA when 7142 and 73147 SNPs were used (Spindel et al., 2015). In common bean, Barili et al. (2018) reported predictive accuracy of 0.40 to 0.47 across Bayesian models based on as fewer as 377 SNP markers. In a recent study, de Sousa et al. (2024) reported that even as few as 500 SSR markers were sufficient for obtaining the required PA in coffee. The requirement of a number of markers as a function of LD has been conclusively demonstrated (Bernardo, 2020). When the number of SNP markers is exceedingly large, *r* ^2^, the measure of LD, was very high (>0.90) to the extent that the adjacent markers became redundant. After redundant markers from among 695 polymorphic SNP markers were removed, 358 markers remained at *r* ^2^ = 0.95, 142 markers at *r* ^2^ = 0.75, and 77 markers at *r* ^2^ = 0.5 (Bernardo, 2020). Hence, the lower number of molecular markers can ideally be used in regular GS‐based breeding in crops like dolichos bean.

### 4.4 Effect of statistical model on the prediction accuracy in dolichos bean

The PA not only varies with crop, TP size, and marker density but also with a statistical model (Zhao et al., 2015). In our study, except for the SPLS model, PA was high for all models. However, the estimates of PA based on rrBLUP were comparable even to those based on complex models like Bayesian, RKHS, RFR, and SVR. On the other hand, nearly 80% of selection candidates were similar between all models, suggesting equivalency of models in selection of better candidates. Our results therefore suggest that it is rather advisable to use rrBLUP for assessing PA in dolichos beans to save resources and computational time. Empirical results also indicated little or no advantage of Bayesian models and even machine learning methods over rrBLUP (Bernardo, 2020).

### 4.5 Effectiveness and efficiency of GS relative to PS

#### 4.5.1 Effectiveness

Early‐generation selection based on the phenotype of especially complexly inherited traits like seed yield is less effective due to low heritability (Bernardo, 2020). The mean seed yield of F_3_ progenies derived from F_2_ plants selected based on genome‐wide marker effects was greater than that of those selected based on PS (Figure 4). These results suggest a higher effectiveness of GS over PS. Significant shifts in marker allele frequency at a greater number of markers in F_2_ plants selected based on predictions than those selected based on PS provide additional evidence for the effectiveness of GS over PS (Figure 5). Significant shifts in marker allele frequency could be attributed to the probable association of these markers with QTL alleles controlling greater seed yield. In response to GS based on the rrBLUP frequency, the alleles shifted at a greater number of loci. Hence, rrBLUP could be considered the best model to implement the GS in dolichos bean.

Significant differences in mean marker allele frequency between GS and F_2_ population across all statistical models highlight the effectiveness of GS, unlike PS, which shows no variation compared to F_2_ BP. The broader distribution of marker allele frequencies in response to GS than PS suggests GS's effectiveness in capturing a wider spectrum of significant alleles. The allele composition of individuals selected based on predicted performance was significantly better compared to genotypes selected based on observed phenotypes. This means that the GS helps in accumulation of favorable alleles in selected candidates. The accumulation of favorable alleles in selection candidates in the F_2_ generation was evidenced by the higher seed yield in the F_3_ progeny generation. These results provide convincing evidence for the effectiveness of GS relative to PS.

#### 4.5.2 Efficiency

To justify the routine use of GS in breeding crops, including dolichos bean, its efficiency also needs to be evaluated. The efficiency of GS relative to PS was quantified based on SD, RS, realized heritability (rh^2^) and realized prediction and phenotypic accuracies (Table 6). The efficiency of GS based on rrBLUP, Bayes A, RKHS, and RFR was greater than that based on other models. Further, the efficiency of GS is almost two times greater than that of PS. A few researchers have also reported greater efficiency of GS relative to PS in different crops. For example, the estimated gains from GS among F_2_ plants were about 90% of the eventual gains from PS for seed yield, moisture content, and 100‐seed weight in maize (Brandariz & Bernardo, 2019; Jacobson et al., 2015).

## 5 CONCLUSION

The greater effectiveness and efficiency of GS relative to PS, even with the low density of SSR markers and the small size of TP, could be explained based on the following three critical factors. These are (i) a small genome size of 367 Mb (Iwata et al., 2013), (ii) a very high level of genetic relatedness between individuals of TP and BP, and (iii) the use of full sibs (FS) as TP. As FS is a closed population derived from parents contrasting for target trait and marker loci, greater LD exists between alleles at QTL controlling the target trait and marker loci. The high level of LD within FS is supported by empirical results that have shown that in maize doubled haploid production, large chromosomal segments or even entire chromosomes are passed intact from parents to offspring (Smith et al., 2008; Sleper & Bernardo, 2017). Therefore, fewer markers are sufficient to achieve a greater PA, and the effectiveness and efficiency of GS are implemented in biparental populations where LD between pairs of markers extends to large genomic regions. Greater efficiency and effectiveness of GS could be further attributed to fewer QTL segregating in TP and BP. This is because both TP and BP are derived from highly related and elite parents. Fewer QTL also results in higher *h* ^2^, as is true in the present study as well. Our results are consistent with those expected from theoretical investigation (Daetwyler et al., 2008; Lian et al., 2014). Our results demonstrate that a low number of markers that cover the entire genome can be best utilized for integrating GS in dolichos bean breeding programs.

## AUTHOR CONTRIBUTIONS

**Mugali Pundalik Kalpana**: Data curation; investigation; methodology; writing—original draft. **Sampangi Ramesh**: Conceptualization; funding acquisition; supervision; writing—review and editing. **Chindi Basavaraj Siddu**: Investigation. **Gonal Basanagouda**: Investigation. **K. Madhusudan**: Resources; writing—review and editing. **Hosakoti Sathish**: Investigation. **Dinesh Sindhu**: Investigation. **Munegowda Kemparaju**: Investigation. **C. Anilkumar**: Conceptualization; data curation; formal analysis; software; writing—review and editing.

## CONFLICT OF INTEREST STATEMENT

The authors declare no conflicts of interest.

## Supporting information



Supplementary Fig. 1a: Line graphs depicting shift in SSR marker allele frequency due to GS and PS relative to F_2_ breeding population (Base population).
Supplementary Fig. 1b: Line graphs depicting shift in SSR marker allele frequency due to GS and PS relative to F_2_ breeding population (Base population).
Supplementary Fig. 1c: Line graphs depicting shift in SSR marker allele frequency due to GS and PS relative to F_2_ breeding population (Base population).

Supplementary Table 1. The assumptions and key features of genomic prediction models used in the study.
Supplementary Table 2: Shifts in frequency of alleles in response to genomic selection and phenotypic selection relative to base population.

## Data Availability

The datasets used and/or analyzed during the current study are available from the corresponding author on reason‐able request. Some of the data are shown in Supporting Information.
