# Supplementary material for: Low density marker‐based effectiveness and efficiency of early‐generation genomic selection relative to phenotype‐based selection in dolichos bean (Lablab purpureus L. Sweet)
Source: Plant Genome. 2025 May 26;18(2):e70039. doi: 10.1002/tpg2.70039 (PMC12107021; doi:10.1002/tpg2.70039)
Supplement: Supplementary file 1 [file TPG2-18-e70039-s001.docx]

Bayes C

SSR marker allelic frequency

| SSR markers | Base | GS | PS |
| --- | --- | --- | --- |
| LPD19 | 0.528169 | 0.642857 | 0.5 |
| LPD36 | 0.474453 | 0.5 | 0.388889 |
| LPD186 | 0.507519 | 0.481481 | 0.481481 |
| LPT10 | 0.482394 | 0.482143 | 0.518519 |
| LPD197 | 0.473881 | 0.464286 | 0.446429 |
| LPT137 | 0.48227 | 0.464286 | 0.446429 |
| LPT192 | 0.44 | 0.442308 | 0.403846 |
| LPT75 | 0.45 | 0.518519 | 0.481481 |
| LPD44 | 0.465812 | 0.5 | 0.434783 |
| LPD79 | 0.470803 | 0.464286 | 0.462963 |
| LPT24 | 0.457692 | 0.303571 | 0.479167 |
| LPT16 | 0.539062 | 0.521739 | 0.5 |
| LPT156 | 0.475694 | 0.678571 | 0.446429 |
| LPD25 | 0.489583 | 0.696429 | 0.464286 |
| LPT157 | 0.482014 | 0.685185 | 0.464286 |
| LPD175 | 0.475524 | 0.678571 | 0.446429 |
| LPT129 | 0.489437 | 0.642857 | 0.462963 |
| LPD190 | 0.532374 | 0.722222 | 0.576923 |
| LPD13 | 0.511278 | 0.615385 | 0.520833 |
| LPT109 | 0.481884 | 0.5 | 0.537037 |
| LPT104 | 0.534615 | 0.583333 | 0.58 |
| LPD201 | 0.485401 | 0.46 | 0.574074 |
| LPD211 | 0.511364 | 0.462963 | 0.62 |
| LPD5 | 0.475177 | 0.446429 | 0.555556 |
| LPD10 | 0.477099 | 0.462963 | 0.56 |
| LPD286 | 0.493056 | 0.732143 | 0.535714 |
| LPD319 | 0.464539 | 0.703704 | 0.5 |
| LPD350 | 0.514286 | 0.767857 | 0.54 |
| LPD38 | 0.492908 | 0.714286 | 0.519231 |
| LPT68 | 0.503546 | 0.722222 | 0.555556 |
| LPD7 | 0.514599 | 0.729167 | 0.535714 |
| LPD18 | 0.518116 | 0.642857 | 0.538462 |
| LPT298 | 0.45122 | 0.5 | 0.6 |
| LPT119 | 0.412698 | 0.42 | 0.4375 |
| LPT249 | 0.467213 | 0.480769 | 0.52381 |
| LPD56 | 0.492308 | 0.462963 | 0.5 |
| LPT182 | 0.51938 | 0.586957 | 0.478261 |
| LPT186 | 0.492754 | 0.557692 | 0.461538 |
| LPD290 | 0.503521 | 0.535714 | 0.518519 |
| LPD304 | 0.450758 | 0.326923 | 0.354167 |
| LPT6 | 0.507143 | 0.303571 | 0.442308 |
| LPD16 | 0.503472 | 0.303571 | 0.428571 |
| LPT173 | 0.510791 | 0.339286 | 0.442308 |
| LPD368 | 0.5 | 0.365385 | 0.416667 |
| LPT146 | 0.518248 | 0.428571 | 0.5 |
| LPT143 | 0.537037 | 0.518519 | 0.535714 |
| LPD287 | 0.514184 | 0.535714 | 0.517857 |
| LPD360 | 0.519231 | 0.458333 | 0.5 |
| LPD37 | 0.492701 | 0.462963 | 0.5625 |
| LPT136 | 0.485915 | 0.464286 | 0.537037 |
| LPD49 | 0.446043 | 0.388889 | 0.576923 |
| LPT257 | 0.428058 | 0.285714 | 0.5 |
| LPT14 | 0.5 | 0.555556 | 0.480769 |
| LPT26 | 0.474359 | 0.56 | 0.47619 |
| LPD157 | 0.437063 | 0.482143 | 0.388889 |
| LPD136 | 0.451128 | 0.423077 | 0.423077 |
| LPT181 | 0.445652 | 0.375 | 0.442308 |
| LPT291 | 0.510417 | 0.589286 | 0.357143 |
| LPD77 | 0.543796 | 0.625 | 0.423077 |
| LPD84 | 0.552448 | 0.625 | 0.446429 |
| LPD135 | 0.559441 | 0.642857 | 0.482143 |
| LPT163 | 0.547445 | 0.5 | 0.481481 |
| LPD178 | 0.527778 | 0.553571 | 0.5 |
| LPT132 | 0.527778 | 0.607143 | 0.5 |
| LPD214 | 0.528169 | 0.642857 | 0.5 |
| LPD243 | 0.543165 | 0.678571 | 0.553571 |
| LPD140 | 0.5 | 0.7 | 0.518519 |
| LPD185 | 0.524476 | 0.517857 | 0.535714 |
| LPD216 | 0.535714 | 0.537037 | 0.611111 |
| LPT183 | 0.519084 | 0.54 | 0.576923 |
| LPD370 | 0.536 | 0.520833 | 0.568182 |
| LPT158 | 0.515504 | 0.434783 | 0.519231 |
| LPT70 | 0.485915 | 0.410714 | 0.446429 |
| LPD260 | 0.485714 | 0.425926 | 0.464286 |
| LPT239 | 0.510563 | 0.464286 | 0.482143 |
| LPD221 | 0.477099 | 0.541667 | 0.423077 |
| LPT189 | 0.482394 | 0.553571 | 0.392857 |
| LPD252 | 0.472222 | 0.65 | 0.34 |
| LPT248 | 0.492908 | 0.648148 | 0.339286 |
| LPD298 | 0.446154 | 0.458333 | 0.3 |
| LPD141 | 0.47037 | 0.481481 | 0.403846 |
| LPT140 | 0.46087 | 0.458333 | 0.423077 |
| LPD352 | 0.473684 | 0.423077 | 0.46 |

Bayes B

SSR marker allelic frequency

| SSR markers | Base | GS | PS |
| --- | --- | --- | --- |
| LPD19 | 0.528169 | 0.607143 | 0.5 |
| LPD36 | 0.474453 | 0.5 | 0.388889 |
| LPD186 | 0.507519 | 0.444444 | 0.481481 |
| LPT10 | 0.482394 | 0.446429 | 0.518519 |
| LPD197 | 0.473881 | 0.444444 | 0.446429 |
| LPT137 | 0.48227 | 0.446429 | 0.446429 |
| LPT192 | 0.44 | 0.403846 | 0.403846 |
| LPT75 | 0.45 | 0.5 | 0.481481 |
| LPD44 | 0.465812 | 0.478261 | 0.434783 |
| LPD79 | 0.470803 | 0.462963 | 0.462963 |
| LPT24 | 0.457692 | 0.303571 | 0.479167 |
| LPT16 | 0.539062 | 0.543478 | 0.5 |
| LPT156 | 0.475694 | 0.660714 | 0.446429 |
| LPD25 | 0.489583 | 0.660714 | 0.464286 |
| LPT157 | 0.482014 | 0.653846 | 0.464286 |
| LPD175 | 0.475524 | 0.642857 | 0.446429 |
| LPT129 | 0.489437 | 0.611111 | 0.462963 |
| LPD190 | 0.532374 | 0.703704 | 0.576923 |
| LPD13 | 0.511278 | 0.634615 | 0.520833 |
| LPT109 | 0.481884 | 0.535714 | 0.537037 |
| LPT104 | 0.534615 | 0.604167 | 0.58 |
| LPD201 | 0.485401 | 0.5 | 0.574074 |
| LPD211 | 0.511364 | 0.518519 | 0.62 |
| LPD5 | 0.475177 | 0.482143 | 0.555556 |
| LPD10 | 0.477099 | 0.462963 | 0.56 |
| LPD286 | 0.493056 | 0.714286 | 0.535714 |
| LPD319 | 0.464539 | 0.685185 | 0.5 |
| LPD350 | 0.514286 | 0.732143 | 0.54 |
| LPD38 | 0.492908 | 0.678571 | 0.519231 |
| LPT68 | 0.503546 | 0.685185 | 0.555556 |
| LPD7 | 0.514599 | 0.6875 | 0.535714 |
| LPD18 | 0.518116 | 0.642857 | 0.538462 |
| LPT298 | 0.45122 | 0.44 | 0.6 |
| LPT119 | 0.412698 | 0.354167 | 0.4375 |
| LPT249 | 0.467213 | 0.423077 | 0.52381 |
| LPD56 | 0.492308 | 0.462963 | 0.5 |
| LPT182 | 0.51938 | 0.586957 | 0.478261 |
| LPT186 | 0.492754 | 0.538462 | 0.461538 |
| LPD290 | 0.503521 | 0.517857 | 0.518519 |
| LPD304 | 0.450758 | 0.288462 | 0.354167 |
| LPT6 | 0.507143 | 0.25 | 0.442308 |
| LPD16 | 0.503472 | 0.25 | 0.428571 |
| LPT173 | 0.510791 | 0.267857 | 0.442308 |
| LPD368 | 0.5 | 0.307692 | 0.416667 |
| LPT146 | 0.518248 | 0.392857 | 0.5 |
| LPT143 | 0.537037 | 0.5 | 0.535714 |
| LPD287 | 0.514184 | 0.5 | 0.517857 |
| LPD360 | 0.519231 | 0.416667 | 0.5 |
| LPD37 | 0.492701 | 0.425926 | 0.5625 |
| LPT136 | 0.485915 | 0.428571 | 0.537037 |
| LPD49 | 0.446043 | 0.388889 | 0.576923 |
| LPT257 | 0.428058 | 0.285714 | 0.5 |
| LPT14 | 0.5 | 0.557692 | 0.480769 |
| LPT26 | 0.474359 | 0.5625 | 0.47619 |
| LPD157 | 0.437063 | 0.517857 | 0.388889 |
| LPD136 | 0.451128 | 0.480769 | 0.423077 |
| LPT181 | 0.445652 | 0.428571 | 0.442308 |
| LPT291 | 0.510417 | 0.607143 | 0.357143 |
| LPD77 | 0.543796 | 0.625 | 0.423077 |
| LPD84 | 0.552448 | 0.625 | 0.446429 |
| LPD135 | 0.559441 | 0.642857 | 0.482143 |
| LPT163 | 0.547445 | 0.537037 | 0.481481 |
| LPD178 | 0.527778 | 0.535714 | 0.5 |
| LPT132 | 0.527778 | 0.589286 | 0.5 |
| LPD214 | 0.528169 | 0.607143 | 0.5 |
| LPD243 | 0.543165 | 0.642857 | 0.553571 |
| LPD140 | 0.5 | 0.66 | 0.518519 |
| LPD185 | 0.524476 | 0.482143 | 0.535714 |
| LPD216 | 0.535714 | 0.518519 | 0.611111 |
| LPT183 | 0.519084 | 0.52 | 0.576923 |
| LPD370 | 0.536 | 0.54 | 0.568182 |
| LPT158 | 0.515504 | 0.456522 | 0.519231 |
| LPT70 | 0.485915 | 0.446429 | 0.446429 |
| LPD260 | 0.485714 | 0.444444 | 0.464286 |
| LPT239 | 0.510563 | 0.5 | 0.482143 |
| LPD221 | 0.477099 | 0.565217 | 0.423077 |
| LPT189 | 0.482394 | 0.607143 | 0.392857 |
| LPD252 | 0.472222 | 0.7 | 0.34 |
| LPT248 | 0.492908 | 0.685185 | 0.339286 |
| LPD298 | 0.446154 | 0.4375 | 0.3 |
| LPD141 | 0.47037 | 0.480769 | 0.403846 |
| LPT140 | 0.46087 | 0.456522 | 0.423077 |
| LPD352 | 0.473684 | 0.403846 | 0.46 |

Bayes A

SSR marker allelic frequency

| SSR markers | Base | GS | PS |
| --- | --- | --- | --- |
| LPD19 | 0.528169 | 0.571429 | 0.5 |
| LPD36 | 0.474453 | 0.464286 | 0.388889 |
| LPD186 | 0.507519 | 0.444444 | 0.481481 |
| LPT10 | 0.482394 | 0.480769 | 0.518519 |
| LPD197 | 0.473881 | 0.464286 | 0.446429 |
| LPT137 | 0.48227 | 0.481481 | 0.446429 |
| LPT192 | 0.44 | 0.480769 | 0.403846 |
| LPT75 | 0.45 | 0.535714 | 0.481481 |
| LPD44 | 0.465812 | 0.5 | 0.434783 |
| LPD79 | 0.470803 | 0.464286 | 0.462963 |
| LPT24 | 0.457692 | 0.392857 | 0.479167 |
| LPT16 | 0.539062 | 0.521739 | 0.5 |
| LPT156 | 0.475694 | 0.696429 | 0.446429 |
| LPD25 | 0.489583 | 0.714286 | 0.464286 |
| LPT157 | 0.482014 | 0.692308 | 0.464286 |
| LPD175 | 0.475524 | 0.696429 | 0.446429 |
| LPT129 | 0.489437 | 0.642857 | 0.462963 |
| LPD190 | 0.532374 | 0.722222 | 0.576923 |
| LPD13 | 0.511278 | 0.66 | 0.520833 |
| LPT109 | 0.481884 | 0.574074 | 0.537037 |
| LPT104 | 0.534615 | 0.625 | 0.58 |
| LPD201 | 0.485401 | 0.538462 | 0.574074 |
| LPD211 | 0.511364 | 0.538462 | 0.62 |
| LPD5 | 0.475177 | 0.5 | 0.555556 |
| LPD10 | 0.477099 | 0.518519 | 0.56 |
| LPD286 | 0.493056 | 0.767857 | 0.535714 |
| LPD319 | 0.464539 | 0.703704 | 0.5 |
| LPD350 | 0.514286 | 0.785714 | 0.54 |
| LPD38 | 0.492908 | 0.75 | 0.519231 |
| LPT68 | 0.503546 | 0.759259 | 0.555556 |
| LPD7 | 0.514599 | 0.75 | 0.535714 |
| LPD18 | 0.518116 | 0.732143 | 0.538462 |
| LPT298 | 0.45122 | 0.54 | 0.6 |
| LPT119 | 0.412698 | 0.375 | 0.4375 |
| LPT249 | 0.467213 | 0.462963 | 0.52381 |
| LPD56 | 0.492308 | 0.425926 | 0.5 |
| LPT182 | 0.51938 | 0.543478 | 0.478261 |
| LPT186 | 0.492754 | 0.519231 | 0.461538 |
| LPD290 | 0.503521 | 0.5 | 0.518519 |
| LPD304 | 0.450758 | 0.346154 | 0.354167 |
| LPT6 | 0.507143 | 0.321429 | 0.442308 |
| LPD16 | 0.503472 | 0.321429 | 0.428571 |
| LPT173 | 0.510791 | 0.339286 | 0.442308 |
| LPD368 | 0.5 | 0.384615 | 0.416667 |
| LPT146 | 0.518248 | 0.428571 | 0.5 |
| LPT143 | 0.537037 | 0.481481 | 0.535714 |
| LPD287 | 0.514184 | 0.482143 | 0.517857 |
| LPD360 | 0.519231 | 0.395833 | 0.5 |
| LPD37 | 0.492701 | 0.407407 | 0.5625 |
| LPT136 | 0.485915 | 0.410714 | 0.537037 |
| LPD49 | 0.446043 | 0.365385 | 0.576923 |
| LPT257 | 0.428058 | 0.321429 | 0.5 |
| LPT14 | 0.5 | 0.555556 | 0.480769 |
| LPT26 | 0.474359 | 0.520833 | 0.47619 |
| LPD157 | 0.437063 | 0.428571 | 0.388889 |
| LPD136 | 0.451128 | 0.42 | 0.423077 |
| LPT181 | 0.445652 | 0.410714 | 0.442308 |
| LPT291 | 0.510417 | 0.535714 | 0.357143 |
| LPD77 | 0.543796 | 0.592593 | 0.423077 |
| LPD84 | 0.552448 | 0.592593 | 0.446429 |
| LPD135 | 0.559441 | 0.607143 | 0.482143 |
| LPT163 | 0.547445 | 0.482143 | 0.481481 |
| LPD178 | 0.527778 | 0.535714 | 0.5 |
| LPT132 | 0.527778 | 0.571429 | 0.5 |
| LPD214 | 0.528169 | 0.571429 | 0.5 |
| LPD243 | 0.543165 | 0.625 | 0.553571 |
| LPD140 | 0.5 | 0.653846 | 0.518519 |
| LPD185 | 0.524476 | 0.535714 | 0.535714 |
| LPD216 | 0.535714 | 0.553571 | 0.611111 |
| LPT183 | 0.519084 | 0.576923 | 0.576923 |
| LPD370 | 0.536 | 0.625 | 0.568182 |
| LPT158 | 0.515504 | 0.586957 | 0.519231 |
| LPT70 | 0.485915 | 0.428571 | 0.446429 |
| LPD260 | 0.485714 | 0.446429 | 0.464286 |
| LPT239 | 0.510563 | 0.5 | 0.482143 |
| LPD221 | 0.477099 | 0.48 | 0.423077 |
| LPT189 | 0.482394 | 0.517857 | 0.392857 |
| LPD252 | 0.472222 | 0.642857 | 0.34 |
| LPT248 | 0.492908 | 0.607143 | 0.339286 |
| LPD298 | 0.446154 | 0.5 | 0.3 |
| LPD141 | 0.47037 | 0.425926 | 0.403846 |
| LPT140 | 0.46087 | 0.403846 | 0.423077 |
| LPD352 | 0.473684 | 0.423077 | 0.46 |

rrBLUP

SSR marker allelic frequency

| SSR markers | Base | GS | PS |
| --- | --- | --- | --- |
| LPD19 | 0.528169 | 0.410714 | 0.5 |
| LPD36 | 0.474453 | 0.519231 | 0.388889 |
| LPD186 | 0.507519 | 0.555556 | 0.481481 |
| LPT10 | 0.482394 | 0.555556 | 0.518519 |
| LPD197 | 0.473881 | 0.596154 | 0.446429 |
| LPT137 | 0.48227 | 0.571429 | 0.446429 |
| LPT192 | 0.44 | 0.586957 | 0.403846 |
| LPT75 | 0.45 | 0.589286 | 0.481481 |
| LPD44 | 0.465812 | 0.543478 | 0.434783 |
| LPD79 | 0.470803 | 0.62963 | 0.462963 |
| LPT24 | 0.457692 | 0.574074 | 0.479167 |
| LPT16 | 0.539062 | 0.479167 | 0.5 |
| LPT156 | 0.475694 | 0.339286 | 0.446429 |
| LPD25 | 0.489583 | 0.321429 | 0.464286 |
| LPT157 | 0.482014 | 0.346154 | 0.464286 |
| LPD175 | 0.475524 | 0.357143 | 0.446429 |
| LPT129 | 0.489437 | 0.410714 | 0.462963 |
| LPD190 | 0.532374 | 0.37037 | 0.576923 |
| LPD13 | 0.511278 | 0.425926 | 0.520833 |
| LPT109 | 0.481884 | 0.288462 | 0.537037 |
| LPT104 | 0.534615 | 0.291667 | 0.58 |
| LPD201 | 0.485401 | 0.307692 | 0.574074 |
| LPD211 | 0.511364 | 0.291667 | 0.62 |
| LPD5 | 0.475177 | 0.357143 | 0.555556 |
| LPD10 | 0.477099 | 0.365385 | 0.56 |
| LPD286 | 0.493056 | 0.267857 | 0.535714 |
| LPD319 | 0.464539 | 0.277778 | 0.5 |
| LPD350 | 0.514286 | 0.166667 | 0.54 |
| LPD38 | 0.492908 | 0.185185 | 0.519231 |
| LPT68 | 0.503546 | 0.166667 | 0.555556 |
| LPD7 | 0.514599 | 0.153846 | 0.535714 |
| LPD18 | 0.518116 | 0.178571 | 0.538462 |
| LPT298 | 0.45122 | 0.5 | 0.6 |
| LPT119 | 0.412698 | 0.659091 | 0.4375 |
| LPT249 | 0.467213 | 0.519231 | 0.52381 |
| LPD56 | 0.492308 | 0.576923 | 0.5 |
| LPT182 | 0.51938 | 0.521739 | 0.478261 |
| LPT186 | 0.492754 | 0.518519 | 0.461538 |
| LPD290 | 0.503521 | 0.482143 | 0.518519 |
| LPD304 | 0.450758 | 0.5 | 0.354167 |
| LPT6 | 0.507143 | 0.589286 | 0.442308 |
| LPD16 | 0.503472 | 0.607143 | 0.428571 |
| LPT173 | 0.510791 | 0.611111 | 0.442308 |
| LPD368 | 0.5 | 0.56 | 0.416667 |
| LPT146 | 0.518248 | 0.518519 | 0.5 |
| LPT143 | 0.537037 | 0.464286 | 0.535714 |
| LPD287 | 0.514184 | 0.464286 | 0.517857 |
| LPD360 | 0.519231 | 0.521739 | 0.5 |
| LPD37 | 0.492701 | 0.518519 | 0.5625 |
| LPT136 | 0.485915 | 0.517857 | 0.537037 |
| LPD49 | 0.446043 | 0.615385 | 0.576923 |
| LPT257 | 0.428058 | 0.607143 | 0.5 |
| LPT14 | 0.5 | 0.428571 | 0.480769 |
| LPT26 | 0.474359 | 0.5 | 0.47619 |
| LPD157 | 0.437063 | 0.607143 | 0.388889 |
| LPD136 | 0.451128 | 0.666667 | 0.423077 |
| LPT181 | 0.445652 | 0.589286 | 0.442308 |
| LPT291 | 0.510417 | 0.482143 | 0.357143 |
| LPD77 | 0.543796 | 0.384615 | 0.423077 |
| LPD84 | 0.552448 | 0.37037 | 0.446429 |
| LPD135 | 0.559441 | 0.357143 | 0.482143 |
| LPT163 | 0.547445 | 0.481481 | 0.481481 |
| LPD178 | 0.527778 | 0.410714 | 0.5 |
| LPT132 | 0.527778 | 0.392857 | 0.5 |
| LPD214 | 0.528169 | 0.410714 | 0.5 |
| LPD243 | 0.543165 | 0.388889 | 0.553571 |
| LPD140 | 0.5 | 0.423077 | 0.518519 |
| LPD185 | 0.524476 | 0.535714 | 0.535714 |
| LPD216 | 0.535714 | 0.407407 | 0.611111 |
| LPT183 | 0.519084 | 0.38 | 0.576923 |
| LPD370 | 0.536 | 0.291667 | 0.568182 |
| LPT158 | 0.515504 | 0.380952 | 0.519231 |
| LPT70 | 0.485915 | 0.517857 | 0.446429 |
| LPD260 | 0.485714 | 0.518519 | 0.464286 |
| LPT239 | 0.510563 | 0.517857 | 0.482143 |
| LPD221 | 0.477099 | 0.545455 | 0.423077 |
| LPT189 | 0.482394 | 0.446429 | 0.392857 |
| LPD252 | 0.472222 | 0.363636 | 0.34 |
| LPT248 | 0.492908 | 0.351852 | 0.339286 |
| LPD298 | 0.446154 | 0.62 | 0.3 |
| LPD141 | 0.47037 | 0.596154 | 0.403846 |
| LPT140 | 0.46087 | 0.62 | 0.423077 |
| LPD352 | 0.473684 | 0.64 | 0.46 |

**Supplementary Fig. 1a: Line graphs depicting shift in SSR marker allele frequency due to GS and PS relative to F_2_ breeding population (Base population)**

SPLS

SSR marker allelic frequency

| SSR markers | Base | GS | PS |
| --- | --- | --- | --- |
| LPD19 | 0.528169 | 0.571429 | 0.5 |
| LPD36 | 0.474453 | 0.537037 | 0.388889 |
| LPD186 | 0.507519 | 0.537037 | 0.481481 |
| LPT10 | 0.482394 | 0.589286 | 0.518519 |
| LPD197 | 0.473881 | 0.553571 | 0.446429 |
| LPT137 | 0.48227 | 0.589286 | 0.446429 |
| LPT192 | 0.44 | 0.52 | 0.403846 |
| LPT75 | 0.45 | 0.576923 | 0.481481 |
| LPD44 | 0.465812 | 0.543478 | 0.434783 |
| LPD79 | 0.470803 | 0.5 | 0.462963 |
| LPT24 | 0.457692 | 0.428571 | 0.479167 |
| LPT16 | 0.539062 | 0.545455 | 0.5 |
| LPT156 | 0.475694 | 0.560714 | 0.446429 |
| LPD25 | 0.489583 | 0.560714 | 0.464286 |
| LPT157 | 0.482014 | 0.56 | 0.464286 |
| LPD175 | 0.475524 | 0.428571 | 0.446429 |
| LPT129 | 0.489437 | 0.62963 | 0.462963 |
| LPD190 | 0.532374 | 0.62963 | 0.576923 |
| LPD13 | 0.511278 | 0.56 | 0.520833 |
| LPT109 | 0.481884 | 0.428571 | 0.537037 |
| LPT104 | 0.534615 | 0.5 | 0.58 |
| LPD201 | 0.485401 | 0.403846 | 0.574074 |
| LPD211 | 0.511364 | 0.44 | 0.62 |
| LPD5 | 0.475177 | 0.339286 | 0.555556 |
| LPD10 | 0.477099 | 0.421429 | 0.56 |
| LPD286 | 0.493056 | 0.714286 | 0.535714 |
| LPD319 | 0.464539 | 0.666667 | 0.5 |
| LPD350 | 0.514286 | 0.732143 | 0.54 |
| LPD38 | 0.492908 | 0.696429 | 0.519231 |
| LPT68 | 0.503546 | 0.696429 | 0.555556 |
| LPD7 | 0.514599 | 0.75 | 0.535714 |
| LPD18 | 0.518116 | 0.703704 | 0.538462 |
| LPT298 | 0.45122 | 0.461538 | 0.6 |
| LPT119 | 0.412698 | 0.442308 | 0.4375 |
| LPT249 | 0.467213 | 0.416667 | 0.52381 |
| LPD56 | 0.492308 | 0.444444 | 0.5 |
| LPT182 | 0.51938 | 0.5 | 0.478261 |
| LPT186 | 0.492754 | 0.461538 | 0.461538 |
| LPD290 | 0.503521 | 0.482143 | 0.518519 |
| LPD304 | 0.450758 | 0.37037 | 0.354167 |
| LPT6 | 0.507143 | 0.267857 | 0.442308 |
| LPD16 | 0.503472 | 0.267857 | 0.428571 |
| LPT173 | 0.510791 | 0.267857 | 0.442308 |
| LPD368 | 0.5 | 0.486923 | 0.416667 |
| LPT146 | 0.518248 | 0.446429 | 0.5 |
| LPT143 | 0.537037 | 0.557692 | 0.535714 |
| LPD287 | 0.514184 | 0.5 | 0.517857 |
| LPD360 | 0.519231 | 0.480769 | 0.5 |
| LPD37 | 0.492701 | 0.481481 | 0.5625 |
| LPT136 | 0.485915 | 0.482143 | 0.537037 |
| LPD49 | 0.446043 | 0.384615 | 0.576923 |
| LPT257 | 0.428058 | 0.303571 | 0.5 |
| LPT14 | 0.5 | 0.52 | 0.480769 |
| LPT26 | 0.474359 | 0.553571 | 0.47619 |
| LPD157 | 0.437063 | 0.446429 | 0.388889 |
| LPD136 | 0.451128 | 0.462963 | 0.423077 |
| LPT181 | 0.445652 | 0.407407 | 0.442308 |
| LPT291 | 0.510417 | 0.589286 | 0.357143 |
| LPD77 | 0.543796 | 0.625 | 0.423077 |
| LPD84 | 0.552448 | 0.625 | 0.446429 |
| LPD135 | 0.559441 | 0.642857 | 0.482143 |
| LPT163 | 0.547445 | 0.535714 | 0.481481 |
| LPD178 | 0.527778 | 0.607143 | 0.5 |
| LPT132 | 0.527778 | 0.589286 | 0.5 |
| LPD214 | 0.528169 | 0.571429 | 0.5 |
| LPD243 | 0.543165 | 0.625 | 0.553571 |
| LPD140 | 0.5 | 0.634615 | 0.518519 |
| LPD185 | 0.524476 | 0.5 | 0.535714 |
| LPD216 | 0.535714 | 0.464286 | 0.611111 |
| LPT183 | 0.519084 | 0.461538 | 0.576923 |
| LPD370 | 0.536 | 0.5 | 0.568182 |
| LPT158 | 0.515504 | 0.5 | 0.519231 |
| LPT70 | 0.485915 | 0.518519 | 0.446429 |
| LPD260 | 0.485714 | 0.517857 | 0.464286 |
| LPT239 | 0.510563 | 0.571429 | 0.482143 |
| LPD221 | 0.477099 | 0.604167 | 0.423077 |
| LPT189 | 0.482394 | 0.592593 | 0.392857 |
| LPD252 | 0.472222 | 0.590909 | 0.34 |
| LPT248 | 0.492908 | 0.589286 | 0.339286 |
| LPD298 | 0.446154 | 0.478261 | 0.3 |
| LPD141 | 0.47037 | 0.444444 | 0.403846 |
| LPT140 | 0.46087 | 0.456522 | 0.423077 |
| LPD352 | 0.473684 | 0.46 | 0.46 |

RKHS

SSR marker allelic frequency

| SSR markers | Base | GS | PS |
| --- | --- | --- | --- |
| LPD19 | 0.528169 | 0.553571 | 0.5 |
| LPD36 | 0.474453 | 0.5 | 0.388889 |
| LPD186 | 0.507519 | 0.444444 | 0.481481 |
| LPT10 | 0.482394 | 0.481481 | 0.518519 |
| LPD197 | 0.473881 | 0.481481 | 0.446429 |
| LPT137 | 0.48227 | 0.482143 | 0.446429 |
| LPT192 | 0.44 | 0.461538 | 0.403846 |
| LPT75 | 0.45 | 0.535714 | 0.481481 |
| LPD44 | 0.465812 | 0.5 | 0.434783 |
| LPD79 | 0.470803 | 0.481481 | 0.462963 |
| LPT24 | 0.457692 | 0.392857 | 0.479167 |
| LPT16 | 0.539062 | 0.543478 | 0.5 |
| LPT156 | 0.475694 | 0.678571 | 0.446429 |
| LPD25 | 0.489583 | 0.678571 | 0.464286 |
| LPT157 | 0.482014 | 0.66 | 0.464286 |
| LPD175 | 0.475524 | 0.660714 | 0.446429 |
| LPT129 | 0.489437 | 0.611111 | 0.462963 |
| LPD190 | 0.532374 | 0.685185 | 0.576923 |
| LPD13 | 0.511278 | 0.6 | 0.520833 |
| LPT109 | 0.481884 | 0.555556 | 0.537037 |
| LPT104 | 0.534615 | 0.62 | 0.58 |
| LPD201 | 0.485401 | 0.519231 | 0.574074 |
| LPD211 | 0.511364 | 0.538462 | 0.62 |
| LPD5 | 0.475177 | 0.482143 | 0.555556 |
| LPD10 | 0.477099 | 0.446429 | 0.56 |
| LPD286 | 0.493056 | 0.732143 | 0.535714 |
| LPD319 | 0.464539 | 0.685185 | 0.5 |
| LPD350 | 0.514286 | 0.75 | 0.54 |
| LPD38 | 0.492908 | 0.714286 | 0.519231 |
| LPT68 | 0.503546 | 0.722222 | 0.555556 |
| LPD7 | 0.514599 | 0.711538 | 0.535714 |
| LPD18 | 0.518116 | 0.714286 | 0.538462 |
| LPT298 | 0.45122 | 0.480769 | 0.6 |
| LPT119 | 0.412698 | 0.354167 | 0.4375 |
| LPT249 | 0.467213 | 0.407407 | 0.52381 |
| LPD56 | 0.492308 | 0.425926 | 0.5 |
| LPT182 | 0.51938 | 0.545455 | 0.478261 |
| LPT186 | 0.492754 | 0.5 | 0.461538 |
| LPD290 | 0.503521 | 0.5 | 0.518519 |
| LPD304 | 0.450758 | 0.307692 | 0.354167 |
| LPT6 | 0.507143 | 0.232143 | 0.442308 |
| LPD16 | 0.503472 | 0.232143 | 0.428571 |
| LPT173 | 0.510791 | 0.232143 | 0.442308 |
| LPD368 | 0.5 | 0.3 | 0.416667 |
| LPT146 | 0.518248 | 0.392857 | 0.5 |
| LPT143 | 0.537037 | 0.5 | 0.535714 |
| LPD287 | 0.514184 | 0.446429 | 0.517857 |
| LPD360 | 0.519231 | 0.38 | 0.5 |
| LPD37 | 0.492701 | 0.388889 | 0.5625 |
| LPT136 | 0.485915 | 0.392857 | 0.537037 |
| LPD49 | 0.446043 | 0.365385 | 0.576923 |
| LPT257 | 0.428058 | 0.303571 | 0.5 |
| LPT14 | 0.5 | 0.5 | 0.480769 |
| LPT26 | 0.474359 | 0.48 | 0.47619 |
| LPD157 | 0.437063 | 0.428571 | 0.388889 |
| LPD136 | 0.451128 | 0.423077 | 0.423077 |
| LPT181 | 0.445652 | 0.410714 | 0.442308 |
| LPT291 | 0.510417 | 0.553571 | 0.357143 |
| LPD77 | 0.543796 | 0.607143 | 0.423077 |
| LPD84 | 0.552448 | 0.607143 | 0.446429 |
| LPD135 | 0.559441 | 0.625 | 0.482143 |
| LPT163 | 0.547445 | 0.517857 | 0.481481 |
| LPD178 | 0.527778 | 0.553571 | 0.5 |
| LPT132 | 0.527778 | 0.589286 | 0.5 |
| LPD214 | 0.528169 | 0.553571 | 0.5 |
| LPD243 | 0.543165 | 0.625 | 0.553571 |
| LPD140 | 0.5 | 0.653846 | 0.518519 |
| LPD185 | 0.524476 | 0.5 | 0.535714 |
| LPD216 | 0.535714 | 0.535714 | 0.611111 |
| LPT183 | 0.519084 | 0.519231 | 0.576923 |
| LPD370 | 0.536 | 0.596154 | 0.568182 |
| LPT158 | 0.515504 | 0.586957 | 0.519231 |
| LPT70 | 0.485915 | 0.410714 | 0.446429 |
| LPD260 | 0.485714 | 0.428571 | 0.464286 |
| LPT239 | 0.510563 | 0.5 | 0.482143 |
| LPD221 | 0.477099 | 0.52 | 0.423077 |
| LPT189 | 0.482394 | 0.589286 | 0.392857 |
| LPD252 | 0.472222 | 0.65 | 0.34 |
| LPT248 | 0.492908 | 0.589286 | 0.339286 |
| LPD298 | 0.446154 | 0.4375 | 0.3 |
| LPD141 | 0.47037 | 0.423077 | 0.403846 |
| LPT140 | 0.46087 | 0.42 | 0.423077 |
| LPD352 | 0.473684 | 0.403846 | 0.46 |

Bayesian Ridge Regression

SSR marker allelic frequency

| SSR markers | Base | GS | PS |
| --- | --- | --- | --- |
| LPD19 | 0.528169 | 0.571429 | 0.5 |
| LPD36 | 0.474453 | 0.5 | 0.388889 |
| LPD186 | 0.507519 | 0.444444 | 0.481481 |
| LPT10 | 0.482394 | 0.482143 | 0.518519 |
| LPD197 | 0.473881 | 0.482143 | 0.446429 |
| LPT137 | 0.48227 | 0.482143 | 0.446429 |
| LPT192 | 0.44 | 0.442308 | 0.403846 |
| LPT75 | 0.45 | 0.518519 | 0.481481 |
| LPD44 | 0.465812 | 0.521739 | 0.434783 |
| LPD79 | 0.470803 | 0.481481 | 0.462963 |
| LPT24 | 0.457692 | 0.351852 | 0.479167 |
| LPT16 | 0.539062 | 0.5 | 0.5 |
| LPT156 | 0.475694 | 0.678571 | 0.446429 |
| LPD25 | 0.489583 | 0.696429 | 0.464286 |
| LPT157 | 0.482014 | 0.673077 | 0.464286 |
| LPD175 | 0.475524 | 0.678571 | 0.446429 |
| LPT129 | 0.489437 | 0.625 | 0.462963 |
| LPD190 | 0.532374 | 0.722222 | 0.576923 |
| LPD13 | 0.511278 | 0.653846 | 0.520833 |
| LPT109 | 0.481884 | 0.517857 | 0.537037 |
| LPT104 | 0.534615 | 0.583333 | 0.58 |
| LPD201 | 0.485401 | 0.48 | 0.574074 |
| LPD211 | 0.511364 | 0.461538 | 0.62 |
| LPD5 | 0.475177 | 0.446429 | 0.555556 |
| LPD10 | 0.477099 | 0.462963 | 0.56 |
| LPD286 | 0.493056 | 0.732143 | 0.535714 |
| LPD319 | 0.464539 | 0.685185 | 0.5 |
| LPD350 | 0.514286 | 0.759259 | 0.54 |
| LPD38 | 0.492908 | 0.711538 | 0.519231 |
| LPT68 | 0.503546 | 0.740741 | 0.555556 |
| LPD7 | 0.514599 | 0.729167 | 0.535714 |
| LPD18 | 0.518116 | 0.685185 | 0.538462 |
| LPT298 | 0.45122 | 0.480769 | 0.6 |
| LPT119 | 0.412698 | 0.4 | 0.4375 |
| LPT249 | 0.467213 | 0.480769 | 0.52381 |
| LPD56 | 0.492308 | 0.462963 | 0.5 |
| LPT182 | 0.51938 | 0.586957 | 0.478261 |
| LPT186 | 0.492754 | 0.557692 | 0.461538 |
| LPD290 | 0.503521 | 0.535714 | 0.518519 |
| LPD304 | 0.450758 | 0.388889 | 0.354167 |
| LPT6 | 0.507143 | 0.303571 | 0.442308 |
| LPD16 | 0.503472 | 0.303571 | 0.428571 |
| LPT173 | 0.510791 | 0.321429 | 0.442308 |
| LPD368 | 0.5 | 0.32 | 0.416667 |
| LPT146 | 0.518248 | 0.392857 | 0.5 |
| LPT143 | 0.537037 | 0.481481 | 0.535714 |
| LPD287 | 0.514184 | 0.5 | 0.517857 |
| LPD360 | 0.519231 | 0.416667 | 0.5 |
| LPD37 | 0.492701 | 0.425926 | 0.5625 |
| LPT136 | 0.485915 | 0.428571 | 0.537037 |
| LPD49 | 0.446043 | 0.384615 | 0.576923 |
| LPT257 | 0.428058 | 0.303571 | 0.5 |
| LPT14 | 0.5 | 0.555556 | 0.480769 |
| LPT26 | 0.474359 | 0.58 | 0.47619 |
| LPD157 | 0.437063 | 0.482143 | 0.388889 |
| LPD136 | 0.451128 | 0.423077 | 0.423077 |
| LPT181 | 0.445652 | 0.375 | 0.442308 |
| LPT291 | 0.510417 | 0.535714 | 0.357143 |
| LPD77 | 0.543796 | 0.589286 | 0.423077 |
| LPD84 | 0.552448 | 0.589286 | 0.446429 |
| LPD135 | 0.559441 | 0.607143 | 0.482143 |
| LPT163 | 0.547445 | 0.5 | 0.481481 |
| LPD178 | 0.527778 | 0.517857 | 0.5 |
| LPT132 | 0.527778 | 0.535714 | 0.5 |
| LPD214 | 0.528169 | 0.571429 | 0.5 |
| LPD243 | 0.543165 | 0.607143 | 0.553571 |
| LPD140 | 0.5 | 0.634615 | 0.518519 |
| LPD185 | 0.524476 | 0.5 | 0.535714 |
| LPD216 | 0.535714 | 0.517857 | 0.611111 |
| LPT183 | 0.519084 | 0.5 | 0.576923 |
| LPD370 | 0.536 | 0.541667 | 0.568182 |
| LPT158 | 0.515504 | 0.5 | 0.519231 |
| LPT70 | 0.485915 | 0.375 | 0.446429 |
| LPD260 | 0.485714 | 0.446429 | 0.464286 |
| LPT239 | 0.510563 | 0.5 | 0.482143 |
| LPD221 | 0.477099 | 0.520833 | 0.423077 |
| LPT189 | 0.482394 | 0.571429 | 0.392857 |
| LPD252 | 0.472222 | 0.666667 | 0.34 |
| LPT248 | 0.492908 | 0.660714 | 0.339286 |
| LPD298 | 0.446154 | 0.4375 | 0.3 |
| LPD141 | 0.47037 | 0.444444 | 0.403846 |
| LPT140 | 0.46087 | 0.4 | 0.423077 |
| LPD352 | 0.473684 | 0.442308 | 0.46 |

Bayesian LASSO

SSR marker allelic frequency

| SSR markers | Base | GS | PS |
| --- | --- | --- | --- |
| LPD19 | 0.528169 | 0.571429 | 0.5 |
| LPD36 | 0.474453 | 0.5 | 0.388889 |
| LPD186 | 0.507519 | 0.444444 | 0.481481 |
| LPT10 | 0.482394 | 0.482143 | 0.518519 |
| LPD197 | 0.473881 | 0.482143 | 0.446429 |
| LPT137 | 0.48227 | 0.482143 | 0.446429 |
| LPT192 | 0.44 | 0.442308 | 0.403846 |
| LPT75 | 0.45 | 0.518519 | 0.481481 |
| LPD44 | 0.465812 | 0.521739 | 0.434783 |
| LPD79 | 0.470803 | 0.481481 | 0.462963 |
| LPT24 | 0.457692 | 0.351852 | 0.479167 |
| LPT16 | 0.539062 | 0.5 | 0.5 |
| LPT156 | 0.475694 | 0.678571 | 0.446429 |
| LPD25 | 0.489583 | 0.696429 | 0.464286 |
| LPT157 | 0.482014 | 0.673077 | 0.464286 |
| LPD175 | 0.475524 | 0.678571 | 0.446429 |
| LPT129 | 0.489437 | 0.625 | 0.462963 |
| LPD190 | 0.532374 | 0.722222 | 0.576923 |
| LPD13 | 0.511278 | 0.653846 | 0.520833 |
| LPT109 | 0.481884 | 0.517857 | 0.537037 |
| LPT104 | 0.534615 | 0.583333 | 0.58 |
| LPD201 | 0.485401 | 0.48 | 0.574074 |
| LPD211 | 0.511364 | 0.461538 | 0.62 |
| LPD5 | 0.475177 | 0.446429 | 0.555556 |
| LPD10 | 0.477099 | 0.462963 | 0.56 |
| LPD286 | 0.493056 | 0.732143 | 0.535714 |
| LPD319 | 0.464539 | 0.685185 | 0.5 |
| LPD350 | 0.514286 | 0.759259 | 0.54 |
| LPD38 | 0.492908 | 0.711538 | 0.519231 |
| LPT68 | 0.503546 | 0.740741 | 0.555556 |
| LPD7 | 0.514599 | 0.729167 | 0.535714 |
| LPD18 | 0.518116 | 0.685185 | 0.538462 |
| LPT298 | 0.45122 | 0.480769 | 0.6 |
| LPT119 | 0.412698 | 0.4 | 0.4375 |
| LPT249 | 0.467213 | 0.480769 | 0.52381 |
| LPD56 | 0.492308 | 0.462963 | 0.5 |
| LPT182 | 0.51938 | 0.586957 | 0.478261 |
| LPT186 | 0.492754 | 0.557692 | 0.461538 |
| LPD290 | 0.503521 | 0.535714 | 0.518519 |
| LPD304 | 0.450758 | 0.388889 | 0.354167 |
| LPT6 | 0.507143 | 0.303571 | 0.442308 |
| LPD16 | 0.503472 | 0.303571 | 0.428571 |
| LPT173 | 0.510791 | 0.321429 | 0.442308 |
| LPD368 | 0.5 | 0.32 | 0.416667 |
| LPT146 | 0.518248 | 0.392857 | 0.5 |
| LPT143 | 0.537037 | 0.481481 | 0.535714 |
| LPD287 | 0.514184 | 0.5 | 0.517857 |
| LPD360 | 0.519231 | 0.416667 | 0.5 |
| LPD37 | 0.492701 | 0.425926 | 0.5625 |
| LPT136 | 0.485915 | 0.428571 | 0.537037 |
| LPD49 | 0.446043 | 0.384615 | 0.576923 |
| LPT257 | 0.428058 | 0.303571 | 0.5 |
| LPT14 | 0.5 | 0.555556 | 0.480769 |
| LPT26 | 0.474359 | 0.58 | 0.47619 |
| LPD157 | 0.437063 | 0.482143 | 0.388889 |
| LPD136 | 0.451128 | 0.423077 | 0.423077 |
| LPT181 | 0.445652 | 0.375 | 0.442308 |
| LPT291 | 0.510417 | 0.535714 | 0.357143 |
| LPD77 | 0.543796 | 0.589286 | 0.423077 |
| LPD84 | 0.552448 | 0.589286 | 0.446429 |
| LPD135 | 0.559441 | 0.607143 | 0.482143 |
| LPT163 | 0.547445 | 0.5 | 0.481481 |
| LPD178 | 0.527778 | 0.517857 | 0.5 |
| LPT132 | 0.527778 | 0.535714 | 0.5 |
| LPD214 | 0.528169 | 0.571429 | 0.5 |
| LPD243 | 0.543165 | 0.607143 | 0.553571 |
| LPD140 | 0.5 | 0.634615 | 0.518519 |
| LPD185 | 0.524476 | 0.5 | 0.535714 |
| LPD216 | 0.535714 | 0.517857 | 0.611111 |
| LPT183 | 0.519084 | 0.5 | 0.576923 |
| LPD370 | 0.536 | 0.541667 | 0.568182 |
| LPT158 | 0.515504 | 0.5 | 0.519231 |
| LPT70 | 0.485915 | 0.375 | 0.446429 |
| LPD260 | 0.485714 | 0.446429 | 0.464286 |
| LPT239 | 0.510563 | 0.5 | 0.482143 |
| LPD221 | 0.477099 | 0.520833 | 0.423077 |
| LPT189 | 0.482394 | 0.571429 | 0.392857 |
| LPD252 | 0.472222 | 0.666667 | 0.34 |
| LPT248 | 0.492908 | 0.660714 | 0.339286 |
| LPD298 | 0.446154 | 0.4375 | 0.3 |
| LPD141 | 0.47037 | 0.444444 | 0.403846 |
| LPT140 | 0.46087 | 0.4 | 0.423077 |
| LPD352 | 0.473684 | 0.442308 | 0.46 |

**Supplementary Fig. 1b: Line graphs depicting shift in SSR marker allele frequency due to GS and PS relative to F_2_ breeding population (Base population)**

**Supplementary Fig. 1c: Line graphs depicting shift in SSR marker allele frequency due to GS and PS relative to F_2_ breeding population (Base population)**

LASSO

SSR marker allelic frequency

| SSR markers | Base | GS | PS |
| --- | --- | --- | --- |
| LPD19 | 0.528169 | 0.571429 | 0.5 |
| LPD36 | 0.474453 | 0.5 | 0.388889 |
| LPD186 | 0.507519 | 0.461538 | 0.481481 |
| LPT10 | 0.482394 | 0.444444 | 0.518519 |
| LPD197 | 0.473881 | 0.425926 | 0.446429 |
| LPT137 | 0.48227 | 0.425926 | 0.446429 |
| LPT192 | 0.44 | 0.403846 | 0.403846 |
| LPT75 | 0.45 | 0.482143 | 0.481481 |
| LPD44 | 0.465812 | 0.4375 | 0.434783 |
| LPD79 | 0.470803 | 0.425926 | 0.462963 |
| LPT24 | 0.457692 | 0.339286 | 0.479167 |
| LPT16 | 0.539062 | 0.547619 | 0.5 |
| LPT156 | 0.475694 | 0.678571 | 0.446429 |
| LPD25 | 0.489583 | 0.678571 | 0.464286 |
| LPT157 | 0.482014 | 0.653846 | 0.464286 |
| LPD175 | 0.475524 | 0.660714 | 0.446429 |
| LPT129 | 0.489437 | 0.589286 | 0.462963 |
| LPD190 | 0.532374 | 0.692308 | 0.576923 |
| LPD13 | 0.511278 | 0.645833 | 0.520833 |
| LPT109 | 0.481884 | 0.482143 | 0.537037 |
| LPT104 | 0.534615 | 0.583333 | 0.58 |
| LPD201 | 0.485401 | 0.480769 | 0.574074 |
| LPD211 | 0.511364 | 0.5 | 0.62 |
| LPD5 | 0.475177 | 0.444444 | 0.555556 |
| LPD10 | 0.477099 | 0.423077 | 0.56 |
| LPD286 | 0.493056 | 0.714286 | 0.535714 |
| LPD319 | 0.464539 | 0.648148 | 0.5 |
| LPD350 | 0.514286 | 0.722222 | 0.54 |
| LPD38 | 0.492908 | 0.678571 | 0.519231 |
| LPT68 | 0.503546 | 0.711538 | 0.555556 |
| LPD7 | 0.514599 | 0.72 | 0.535714 |
| LPD18 | 0.518116 | 0.666667 | 0.538462 |
| LPT298 | 0.45122 | 0.5 | 0.6 |
| LPT119 | 0.412698 | 0.395833 | 0.4375 |
| LPT249 | 0.467213 | 0.458333 | 0.52381 |
| LPD56 | 0.492308 | 0.423077 | 0.5 |
| LPT182 | 0.51938 | 0.565217 | 0.478261 |
| LPT186 | 0.492754 | 0.54 | 0.461538 |
| LPD290 | 0.503521 | 0.518519 | 0.518519 |
| LPD304 | 0.450758 | 0.34 | 0.354167 |
| LPT6 | 0.507143 | 0.214286 | 0.442308 |
| LPD16 | 0.503472 | 0.214286 | 0.428571 |
| LPT173 | 0.510791 | 0.232143 | 0.442308 |
| LPD368 | 0.5 | 0.288462 | 0.416667 |
| LPT146 | 0.518248 | 0.428571 | 0.5 |
| LPT143 | 0.537037 | 0.5 | 0.535714 |
| LPD287 | 0.514184 | 0.464286 | 0.517857 |
| LPD360 | 0.519231 | 0.4375 | 0.5 |
| LPD37 | 0.492701 | 0.442308 | 0.5625 |
| LPT136 | 0.485915 | 0.444444 | 0.537037 |
| LPD49 | 0.446043 | 0.423077 | 0.576923 |
| LPT257 | 0.428058 | 0.339286 | 0.5 |
| LPT14 | 0.5 | 0.56 | 0.480769 |
| LPT26 | 0.474359 | 0.590909 | 0.47619 |
| LPD157 | 0.437063 | 0.517857 | 0.388889 |
| LPD136 | 0.451128 | 0.5 | 0.423077 |
| LPT181 | 0.445652 | 0.464286 | 0.442308 |
| LPT291 | 0.510417 | 0.625 | 0.357143 |
| LPD77 | 0.543796 | 0.62963 | 0.423077 |
| LPD84 | 0.552448 | 0.62963 | 0.446429 |
| LPD135 | 0.559441 | 0.642857 | 0.482143 |
| LPT163 | 0.547445 | 0.462963 | 0.481481 |
| LPD178 | 0.527778 | 0.517857 | 0.5 |
| LPT132 | 0.527778 | 0.589286 | 0.5 |
| LPD214 | 0.528169 | 0.571429 | 0.5 |
| LPD243 | 0.543165 | 0.642857 | 0.553571 |
| LPD140 | 0.5 | 0.64 | 0.518519 |
| LPD185 | 0.524476 | 0.535714 | 0.535714 |
| LPD216 | 0.535714 | 0.5 | 0.611111 |
| LPT183 | 0.519084 | 0.519231 | 0.576923 |
| LPD370 | 0.536 | 0.6 | 0.568182 |
| LPT158 | 0.515504 | 0.5625 | 0.519231 |
| LPT70 | 0.485915 | 0.535714 | 0.446429 |
| LPD260 | 0.485714 | 0.518519 | 0.464286 |
| LPT239 | 0.510563 | 0.571429 | 0.482143 |
| LPD221 | 0.477099 | 0.613636 | 0.423077 |
| LPT189 | 0.482394 | 0.642857 | 0.392857 |
| LPD252 | 0.472222 | 0.690476 | 0.34 |
| LPT248 | 0.492908 | 0.648148 | 0.339286 |
| LPD298 | 0.446154 | 0.395833 | 0.3 |
| LPD141 | 0.47037 | 0.423077 | 0.403846 |
| LPT140 | 0.46087 | 0.395833 | 0.423077 |
| LPD352 | 0.473684 | 0.444444 | 0.46 |

SVR

SSR marker allelic frequency

| SSR markers | Base | GS | PS |
| --- | --- | --- | --- |
| LPD19 | 0.528169 | 0.464286 | 0.5 |
| LPD36 | 0.474453 | 0.553571 | 0.388889 |
| LPD186 | 0.507519 | 0.553571 | 0.481481 |
| LPT10 | 0.482394 | 0.553571 | 0.518519 |
| LPD197 | 0.473881 | 0.589286 | 0.446429 |
| LPT137 | 0.48227 | 0.589286 | 0.446429 |
| LPT192 | 0.44 | 0.660714 | 0.403846 |
| LPT75 | 0.45 | 0.5 | 0.481481 |
| LPD44 | 0.465812 | 0.456522 | 0.434783 |
| LPD79 | 0.470803 | 0.481481 | 0.462963 |
| LPT24 | 0.457692 | 0.538462 | 0.479167 |
| LPT16 | 0.539062 | 0.56 | 0.5 |
| LPT156 | 0.475694 | 0.321429 | 0.446429 |
| LPD25 | 0.489583 | 0.303571 | 0.464286 |
| LPT157 | 0.482014 | 0.3125 | 0.464286 |
| LPD175 | 0.475524 | 0.285714 | 0.446429 |
| LPT129 | 0.489437 | 0.333333 | 0.462963 |
| LPD190 | 0.532374 | 0.277778 | 0.576923 |
| LPD13 | 0.511278 | 0.384615 | 0.520833 |
| LPT109 | 0.481884 | 0.518519 | 0.537037 |
| LPT104 | 0.534615 | 0.388889 | 0.58 |
| LPD201 | 0.485401 | 0.480769 | 0.574074 |
| LPD211 | 0.511364 | 0.462963 | 0.62 |
| LPD5 | 0.475177 | 0.535714 | 0.555556 |
| LPD10 | 0.477099 | 0.571429 | 0.56 |
| LPD286 | 0.493056 | 0.321429 | 0.535714 |
| LPD319 | 0.464539 | 0.375 | 0.5 |
| LPD350 | 0.514286 | 0.351852 | 0.54 |
| LPD38 | 0.492908 | 0.384615 | 0.519231 |
| LPT68 | 0.503546 | 0.351852 | 0.555556 |
| LPD7 | 0.514599 | 0.365385 | 0.535714 |
| LPD18 | 0.518116 | 0.351852 | 0.538462 |
| LPT298 | 0.45122 | 0.615385 | 0.6 |
| LPT119 | 0.412698 | 0.666667 | 0.4375 |
| LPT249 | 0.467213 | 0.645833 | 0.52381 |
| LPD56 | 0.492308 | 0.596154 | 0.5 |
| LPT182 | 0.51938 | 0.479167 | 0.478261 |
| LPT186 | 0.492754 | 0.518519 | 0.461538 |
| LPD290 | 0.503521 | 0.517857 | 0.518519 |
| LPD304 | 0.450758 | 0.54 | 0.354167 |
| LPT6 | 0.507143 | 0.607143 | 0.442308 |
| LPD16 | 0.503472 | 0.625 | 0.428571 |
| LPT173 | 0.510791 | 0.611111 | 0.442308 |
| LPD368 | 0.5 | 0.583333 | 0.416667 |
| LPT146 | 0.518248 | 0.666667 | 0.5 |
| LPT143 | 0.537037 | 0.576923 | 0.535714 |
| LPD287 | 0.514184 | 0.625 | 0.517857 |
| LPD360 | 0.519231 | 0.666667 | 0.5 |
| LPD37 | 0.492701 | 0.696429 | 0.5625 |
| LPT136 | 0.485915 | 0.696429 | 0.537037 |
| LPD49 | 0.446043 | 0.722222 | 0.576923 |
| LPT257 | 0.428058 | 0.767857 | 0.5 |
| LPT14 | 0.5 | 0.576923 | 0.480769 |
| LPT26 | 0.474359 | 0.586957 | 0.47619 |
| LPD157 | 0.437063 | 0.642857 | 0.388889 |
| LPD136 | 0.451128 | 0.730769 | 0.423077 |
| LPT181 | 0.445652 | 0.714286 | 0.442308 |
| LPT291 | 0.510417 | 0.571429 | 0.357143 |
| LPD77 | 0.543796 | 0.537037 | 0.423077 |
| LPD84 | 0.552448 | 0.517857 | 0.446429 |
| LPD135 | 0.559441 | 0.517857 | 0.482143 |
| LPT163 | 0.547445 | 0.5 | 0.481481 |
| LPD178 | 0.527778 | 0.5 | 0.5 |
| LPT132 | 0.527778 | 0.553571 | 0.5 |
| LPD214 | 0.528169 | 0.535714 | 0.5 |
| LPD243 | 0.543165 | 0.482143 | 0.553571 |
| LPD140 | 0.5 | 0.480769 | 0.518519 |
| LPD185 | 0.524476 | 0.555556 | 0.535714 |
| LPD216 | 0.535714 | 0.446429 | 0.611111 |
| LPT183 | 0.519084 | 0.5 | 0.576923 |
| LPD370 | 0.536 | 0.446429 | 0.568182 |
| LPT158 | 0.515504 | 0.403846 | 0.519231 |
| LPT70 | 0.485915 | 0.611111 | 0.446429 |
| LPD260 | 0.485714 | 0.535714 | 0.464286 |
| LPT239 | 0.510563 | 0.5 | 0.482143 |
| LPD221 | 0.477099 | 0.555556 | 0.423077 |
| LPT189 | 0.482394 | 0.517857 | 0.392857 |
| LPD252 | 0.472222 | 0.456522 | 0.34 |
| LPT248 | 0.492908 | 0.5 | 0.339286 |
| LPD298 | 0.446154 | 0.692308 | 0.3 |
| LPD141 | 0.47037 | 0.589286 | 0.403846 |
| LPT140 | 0.46087 | 0.615385 | 0.423077 |
| LPD352 | 0.473684 | 0.56 | 0.46 |

RFR

SSR marker allelic frequency

| SSR markers | Base | GS | PS |
| --- | --- | --- | --- |
| LPD19 | 0.528169 | 0.410714 | 0.5 |
| LPD36 | 0.474453 | 0.519231 | 0.388889 |
| LPD186 | 0.507519 | 0.5 | 0.481481 |
| LPT10 | 0.482394 | 0.481481 | 0.518519 |
| LPD197 | 0.473881 | 0.462963 | 0.446429 |
| LPT137 | 0.48227 | 0.482143 | 0.446429 |
| LPT192 | 0.44 | 0.557692 | 0.403846 |
| LPT75 | 0.45 | 0.5 | 0.481481 |
| LPD44 | 0.465812 | 0.456522 | 0.434783 |
| LPD79 | 0.470803 | 0.480769 | 0.462963 |
| LPT24 | 0.457692 | 0.480769 | 0.479167 |
| LPT16 | 0.539062 | 0.54 | 0.5 |
| LPT156 | 0.475694 | 0.375 | 0.446429 |
| LPD25 | 0.489583 | 0.375 | 0.464286 |
| LPT157 | 0.482014 | 0.403846 | 0.464286 |
| LPD175 | 0.475524 | 0.428571 | 0.446429 |
| LPT129 | 0.489437 | 0.446429 | 0.462963 |
| LPD190 | 0.532374 | 0.442308 | 0.576923 |
| LPD13 | 0.511278 | 0.5 | 0.520833 |
| LPT109 | 0.481884 | 0.703704 | 0.537037 |
| LPT104 | 0.534615 | 0.52 | 0.58 |
| LPD201 | 0.485401 | 0.703704 | 0.574074 |
| LPD211 | 0.511364 | 0.673077 | 0.62 |
| LPD5 | 0.475177 | 0.703704 | 0.555556 |
| LPD10 | 0.477099 | 0.730769 | 0.56 |
| LPD286 | 0.493056 | 0.375 | 0.535714 |
| LPD319 | 0.464539 | 0.388889 | 0.5 |
| LPD350 | 0.514286 | 0.333333 | 0.54 |
| LPD38 | 0.492908 | 0.388889 | 0.519231 |
| LPT68 | 0.503546 | 0.346154 | 0.555556 |
| LPD7 | 0.514599 | 0.375 | 0.535714 |
| LPD18 | 0.518116 | 0.346154 | 0.538462 |
| LPT298 | 0.45122 | 0.462963 | 0.6 |
| LPT119 | 0.412698 | 0.46 | 0.4375 |
| LPT249 | 0.467213 | 0.478261 | 0.52381 |
| LPD56 | 0.492308 | 0.44 | 0.5 |
| LPT182 | 0.51938 | 0.521739 | 0.478261 |
| LPT186 | 0.492754 | 0.538462 | 0.461538 |
| LPD290 | 0.503521 | 0.425926 | 0.518519 |
| LPD304 | 0.450758 | 0.557692 | 0.354167 |
| LPT6 | 0.507143 | 0.62963 | 0.442308 |
| LPD16 | 0.503472 | 0.625 | 0.428571 |
| LPT173 | 0.510791 | 0.625 | 0.442308 |
| LPD368 | 0.5 | 0.642857 | 0.416667 |
| LPT146 | 0.518248 | 0.685185 | 0.5 |
| LPT143 | 0.537037 | 0.634615 | 0.535714 |
| LPD287 | 0.514184 | 0.678571 | 0.517857 |
| LPD360 | 0.519231 | 0.72 | 0.5 |
| LPD37 | 0.492701 | 0.759259 | 0.5625 |
| LPT136 | 0.485915 | 0.759259 | 0.537037 |
| LPD49 | 0.446043 | 0.685185 | 0.576923 |
| LPT257 | 0.428058 | 0.759259 | 0.5 |
| LPT14 | 0.5 | 0.42 | 0.480769 |
| LPT26 | 0.474359 | 0.478261 | 0.47619 |
| LPD157 | 0.437063 | 0.553571 | 0.388889 |
| LPD136 | 0.451128 | 0.5 | 0.423077 |
| LPT181 | 0.445652 | 0.517857 | 0.442308 |
| LPT291 | 0.510417 | 0.464286 | 0.357143 |
| LPD77 | 0.543796 | 0.464286 | 0.423077 |
| LPD84 | 0.552448 | 0.464286 | 0.446429 |
| LPD135 | 0.559441 | 0.446429 | 0.482143 |
| LPT163 | 0.547445 | 0.425926 | 0.481481 |
| LPD178 | 0.527778 | 0.392857 | 0.5 |
| LPT132 | 0.527778 | 0.392857 | 0.5 |
| LPD214 | 0.528169 | 0.410714 | 0.5 |
| LPD243 | 0.543165 | 0.375 | 0.553571 |
| LPD140 | 0.5 | 0.403846 | 0.518519 |
| LPD185 | 0.524476 | 0.464286 | 0.535714 |
| LPD216 | 0.535714 | 0.464286 | 0.611111 |
| LPT183 | 0.519084 | 0.5 | 0.576923 |
| LPD370 | 0.536 | 0.442308 | 0.568182 |
| LPT158 | 0.515504 | 0.46 | 0.519231 |
| LPT70 | 0.485915 | 0.574074 | 0.446429 |
| LPD260 | 0.485714 | 0.537037 | 0.464286 |
| LPT239 | 0.510563 | 0.446429 | 0.482143 |
| LPD221 | 0.477099 | 0.458333 | 0.423077 |
| LPT189 | 0.482394 | 0.464286 | 0.392857 |
| LPD252 | 0.472222 | 0.54 | 0.34 |
| LPT248 | 0.492908 | 0.517857 | 0.339286 |
| LPD298 | 0.446154 | 0.576923 | 0.3 |
| LPD141 | 0.47037 | 0.571429 | 0.403846 |
| LPT140 | 0.46087 | 0.583333 | 0.423077 |
| LPD352 | 0.473684 | 0.462963 | 0.46 |
