# Supplementary material for: Low density marker‐based effectiveness and efficiency of early‐generation genomic selection relative to phenotype‐based selection in dolichos bean (Lablab purpureus L. Sweet)
Source: Plant Genome. 2025 May 26;18(2):e70039. doi: 10.1002/tpg2.70039 (PMC12107021; doi:10.1002/tpg2.70039)
Supplement: Supplementary file 1 — Supplementary Fig. 1a: Line graphs depicting shift in SSR marker allele frequency due to GS and PS relative to F2 breeding population (Base population). Supplementary Fig. 1b: Line graphs depicting shift in SSR marker allele frequency due to GS and PS relative to F2 breeding population (Base population). Supplementary Fig. 1c: Line graphs depicting shift in SSR marker allele frequency due to GS and PS relative to F2 breeding population (Base population). [file TPG2-18-e70039-s001.docx]

**Supplementary Fig. 1a: Line graphs depicting shift in SSR marker allele frequency due to GS and PS relative to F_2_ breeding population (Base population)**

**Supplementary Fig. 1b: Line graphs depicting shift in SSR marker allele frequency due to GS and PS relative to F_2_ breeding population (Base population)**

**Supplementary Fig. 1c: Line graphs depicting shift in SSR marker allele frequency due to GS and PS relative to F_2_ breeding population (Base population)**
