# Supplementary material for: Low density marker‐based effectiveness and efficiency of early‐generation genomic selection relative to phenotype‐based selection in dolichos bean (Lablab purpureus L. Sweet)
Source: Plant Genome. 2025 May 26;18(2):e70039. doi: 10.1002/tpg2.70039 (PMC12107021; doi:10.1002/tpg2.70039)
Supplement: Supplementary file 2 — Supplementary Table 1. The assumptions and key features of genomic prediction models used in the study. Supplementary Table 2: Shifts in frequency of alleles in response to genomic selection and phenotypic selection relative to base population. [file TPG2-18-e70039-s002.docx]

**Supplementary Table 1.** The assumptions and key features of genomic prediction models used in the study

| Model | **Models/components** | **Assumptions** | **Key features** |
| --- | --- | --- | --- |
| Bayes A | β_j_ ~ t (d fβ, Sβ)  β_j_, is the additive effect of the j^th^; t, scaled-t distribution; d fβ, degree of freedom; Sβ, scale parameters; | - The Bayesian models do not assume a normal distribution of marker effects. Instead, they assume that few markers will have large effects on the trait, allowing markers to have different effects and variances. - All marker effects are assumed to be independently and identically distributed. | - Utilizes an inverse chi-square (χ^2^) on marker variances yielding a scaled t-distribution for marker effects. - It shrinks tiny marker effects towards zero and larger values survive. - Accounts for non-additive interactions/effects |
| Bayes B | β_j_ = $\left\{ \begin{aligned} 1/2\gamma\text{ λ exp(-λ}\left\vert\beta_{j} \right\vert for \beta j \neq0 \\ \left( 1-\gamma\right) for \beta j=0 \end{aligned} \right.$  β_j_, is the additive effect of the j^th^; t, scaled-t distribution; d fβ, degree of freedom; Sβ, scale parameters;  $\text{λ}$, fraction of the SSR that are in linkage disequilibrium with a quantitative trait locus;  p, probability of the marker effect equal to zero; DE, double exponential;  l, parameter of exponential distribution. |  | - Similar to Bayes A, uses an inverse χ^2^ resulting in scaled t-distribution. - Unlike Bayes A, utilizes both shrinkage and variable selection. - Accounts for non-additive interactions/effects |
| Bayes C | $\left. \beta_{j} \right\vert\pi, \sigma_{\beta_{j}}^{2}\left\{ \begin{aligned} \beta_{j\sim0} with prob \pi\\ \beta_{j \sim N (0, \sigma_{\beta_{J}}^{2})} with prob (1-\pi) \end{aligned} \right.$  β_j_, is the additive effect of the j^th^; t, scaled-t distribution; d fβ, degree of freedom; Sβ, scale parameters;  $\text{λ}$, fraction of the SSR that are in linkage disequilibrium with a quantitative trait locus;  $\pi$, probability of the marker effect equal to zero;  DE, double exponential;  l, parameter of exponential distribution. |  | - Applies both shrinkage and variable selection methods - Characterized by a Gaussian distribution. - Accounts for non-additive interactions |
| Bayesian LASSO | β_j_ ~ *DE* ($\text{λ}^{2}$ , $\sigma_{e}^{2}$)  β_j_, is the additive effect of the j^th^; t, scaled-t distribution;  $\text{λ}$, fraction of the SSR that are in linkage disequilibrium with a quantitative trait locus;  $\pi$, probability of the marker effect equal to zero;  DE, double exponential;  $\sigma_{e}^{2}$ : Genetic varainces |  | - Bayesian LASSO uses the Laplace (double exponential, DE) distribution, where the prior assigned to marker effects. This prior assigns the same variance or prior uncertainty to all marker effects. - Removes markers from the model, contrary to what happens in variable selection approaches. - Shrink effects more strongly toward zero than the Gaussian prior. - Accounts for non-additive interactions/effects |
| Bayesian Ridge Regression | $a_{i} \left\vert\sigma_{a}^{2} \sim N\left( 0,\sigma_{a}^{2} \right); \sigma_{a}^{2} \right\vert v_{a}, S_{a}\sim\chi^{-2}(v_{a},S_{a})$    Or  $Yi=\mu+\sum_{j=1}^{p} xj\beta j+e$  Yi = phenotype of the i^th^ individual  xj = genotype of the individual at the j^th^ marker  $\beta j$ is the allelic substitution effect for the j^th^ marker and e_i_ is the random residual |  | - Induces homogeneous shrinkage of all marker effects towards zero and yields a Gaussian distribution of marker effects. - Similar to RR-BLUP, there is a problem of QTL linkages to the marker - Accounts for non-additive interactions/effects |
| Reduced Kernel Hilbert Space (RKHS) | $Y=W\mu+K_{h}\alpha= \varepsilon$  where μ is a vector of fixed effects and ε is a vector of random residuals. The parameters α and ε are assumed to have independent prior distributions α ∼ N (0, K_h_) and ε ∼ N (0, I), respectively. Matrix K_h_ depends on a reproducing kernel function with a smoothing parameter h | Assumes a *priori* definition of kernel function | - The RKHS approach first uses a kernel function to convert the marker data set into a set of distances between pairs of observations that results in a square matrix to be used in a linear model. Because RKHS regression does not assume linearity, it might capture non additive effects. - Based on genetic distance and a kernel function with a smoothing parameter to regulate the distribution of QTL effects. - Effective for detecting nonadditive gene effects. |
| Sparse Partial Least Square (SPLS) | X = TC^T^ + E_X_,  Y = SD^T^ + Ey,  T and S are matrices of estimated latent features called X‐scores, and Y‐scores, C and D are matrices of *X‐loadings* and *Y‐loadings*, and Ex and Ey are residual matrices |  | - SPLS is a form of partial least square regression |
| Ridge Regression BLUP  (rrBLUP) | P=1β + Zm + ε  Where,  'P' is the vector of phenotypes from the additive genetic values  'B' is the overall mean fitted as a fixed effect,  'm' is the vector of random marker effects  'e' is the vector of random error effects,  '1' is a vector of ones  'Z' is the incidence matrix | Assumes markers have equal variances with small but non-zero effects. Some QTL are in LD with marker loci, whereas others are not | - It is a shrinkage method to obtain GEBVs by incorporating genomic information into BLUP using ridge regression. - Computed from a realized-relation matrix based on markers |
| LASSO | $Yi=\sum_{j=1}^{m} xij\beta j+ei$  Where,  Yi = phenotype of the i^th^ individual  xij = genotype of the i^th^ individual at the j^th^ marker of 1 to m markers, with x_ij_=0 for genotype 11 xij=1 for genotype 12 and  xij=2 for genotype 22  $\beta j$ is the allelic substitution effect for the j^th^ marker and e_i_ is the random residual of the i^th^ individual | Assumes that many chromosome segments will not contain quantitative trait locus (QTL) and therefore have zero effect, and only few are real QTLs | - The model includes only a subset of explanatory variables, setting to zero those that have nil effects. - Accounts for non-additive interactions/effects |
| Support Vector Regression (SVR) | $f (x) = w/x + b$  Where,  w is a vector of unknown weights (i.e., regression coefficients) and b is the bias. | Assumption free | - Support vector regression (SVR) is an application of the support vector machine (SVM). The objective of SVM is to find the best hyperplane with the maximal margin in a genotypic matrix with respect to a phenotypic value and predict the correct classification/ regression of unseen examples. - Accounts for non-additive interactions |
| Random Forest Regression (RFR) | $\overset{^}{\overbrace{f}}\begin{matrix} B \\ rf \end{matrix}(x)= \frac{1}{B} \sum_{b=1}^{B} T(x, \Psi b)$  Where,  B is trees, Ψ *_b_* characterizes the *b*^th^ RF tree in terms of split variables, cut points at each node, and terminal node values | Assumption free | - The RF method is a machine learning model based on the identification of an objective function. The objective function measures the distance between the RF output and desired scores to modify internal parameters to reduce this error. - In genomic prediction it randomly draws sample from training data and forms bootstrap samples, which are considered as a tree. Each tree generates subsamples known as nodes. The final prediction is calculated as the average values over all the trees. - The RF model attempts to reduce the computational cost to train the model, capture complex interactions and reduce the over-fitting risk in the data. - Accounts for non-additive interactions/effects |

**Supplementary Table 2: Shifts in frequency of alleles in response to genomic selection and phenotypic selection relative to base population**

| **rrBLUP** | | | | | | | | **Bayes A** | | | | | | | |
| --- | --- | --- | --- | --- | --- | --- | --- | --- | --- | --- | --- | --- | --- | --- | --- |
| **Sl. No** | **Markers** | **Base Population**  **(F_2_ BP)** | | **GEBV based Selected individuals** | | **‘t’ statistic** | **‘P’ value** | **Sl. No** | **Markers** | **Base Population**  **(F_2_ BP)** | | **GEBV based Selected individuals** | | **‘t’ statistic** | **‘P’ value** |
|  |  | **HA 5** | **HA 10-8** | **HA 5** | **HA 10-8** |  |  |  |  | **HA 5** | **HA 10-8** | **HA 5** | **HA 10-8** |  |  |
| 01 | LPD7 | 0.51 | 0.49 | 0.15 | 0.85 | 4.94 | 4.16 × 10^-6^ | 01 | LPD286 | 0.46 | 0.54 | 0.77 | 0.23 | -3.76 | 4.30 × 10^-4^ |
| 02 | LPD350 | 0.51 | 0.49 | 0.17 | 0.83 | 4.76 | 8.99 × 10^-6^ | 02 | LPD350 | 0.51 | 0.49 | 0.79 | 0.21 | -3.72 | 5.00 × 10^-4^ |
| 03 | LPD18 | 0.52 | 0.48 | 0.18 | 0.82 | 4.65 | 1.43 × 10^-5^ | 03 | LPD38 | 0.49 | 0.51 | 0.75 | 0.25 | -3.52 | 9.70 × 10^-4^ |
| 04 | LPT68 | 0.50 | 0.50 | 0.17 | 0.83 | 4.61 | 1.66 × 10^-5^ | 04 | LPT68 | 0.49 | 0.51 | 0.76 | 0.24 | -3.50 | 1.03 × 10^-3^ |
| 05 | LPD38 | 0.49 | 0.51 | 0.19 | 0.81 | 4.21 | 1.56 × 10^-3^ | 05 | LPD319 | 0.50 | 0.50 | 0.70 | 0.30 | -3.28 | 2.13 × 10^-3^ |
| 06 | LPT119 | 0.41 | 0.59 | 0.66 | 0.34 | -3.37 | 1.70 × 10^-3^ | 06 | LPD7 | 0.48 | 0.52 | 0.75 | 0.25 | -3.22 | 2.50 × 10^-3^ |
| 07 | LPD370 | 0.54 | 0.46 | 0.29 | 0.71 | 3.35 | 1.81 × 10^-3^ | 07 | LPD25 | 0.48 | 0.52 | 0.71 | 0.29 | -3.08 | 3.87 × 10^-3^ |
| 08 | LPT104 | 0.53 | 0.47 | 0.29 | 0.71 | 3.33 | 3.70 × 10^-2^ | 08 | LPD175 | 0.54 | 0.46 | 0.70 | 0.30 | -3.03 | 4.50 × 10^-3^ |
| 09 | LPD286 | 0.49 | 0.51 | 0.27 | 0.73 | 3.08 | 4.72 × 10^-3^ | 09 | LPT156 | 0.51 | 0.49 | 0.70 | 0.30 | -3.02 | 4.53 × 10^-3^ |
| 10 | LPD211 | 0.51 | 0.49 | 0.29 | 0.71 | 3.01 | 0.005 | 10 | LPD18 | 0.49 | 0.51 | 0.73 | 0.27 | -2.93 | 5.89 × 10^-3^ |
| 11 | LPD136 | 0.45 | 0.55 | 0.67 | 0.33 | -2.95 | 0.006 | 11 | LPT157 | 0.49 | 0.51 | 0.69 | 0.31 | -2.88 | 6.79 × 10^-3^ |
| 12 | LPD135 | 0.56 | 0.44 | 0.36 | 0.64 | 2.77 | 0.009 | 12 | LPD190 | 0.45 | 0.55 | 0.72 | 0.28 | -2.60 | 1.42 × 10^-2^ |
| 13 | LPT109 | 0.48 | 0.52 | 0.29 | 0.71 | 2.65 | 0.013 | 13 | LPT6 | 0.51 | 0.49 | 0.32 | 0.68 | 2.54 | 1.63 × 10^-2^ |
| 14 | LPD319 | 0.46 | 0.54 | 0.28 | 0.72 | 2.56 | 0.016 | 14 | LPD16 | 0.50 | 0.50 | 0.32 | 0.68 | 2.49 | 1.84 × 10^-2^ |
| 15 | LPD84 | 0.55 | 0.45 | 0.37 | 0.63 | 2.49 | 0.018 | 15 | LPT173 | 0.48 | 0.52 | 0.34 | 0.66 | 2.35 | 2.60 × 10^-2^ |
| 16 | LPT257 | 0.43 | 0.57 | 0.61 | 0.39 | -2.45 | 0.020 | 16 | LPD252 | 0.54 | 0.46 | 0.64 | 0.36 | -2.34 | 2.66 × 10^-2^ |
| 17 | LPD201 | 0.49 | 0.51 | 0.31 | 0.69 | 2.43 | 0.021 | 17 | LPD140 | 0.48 | 0.52 | 0.65 | 0.35 | -2.11 | 4.39 × 10^-2^ |
| 18 | LPD298 | 0.45 | 0.55 | 0.62 | 0.38 | -2.38 | 0.024 | 18 | LPT129 | 0.53 | 0.47 | 0.64 | 0.36 | -2.10 | 4.45 × 10^-2^ |
| 19 | LPD157 | 0.44 | 0.56 | 0.61 | 0.39 | -2.33 | 0.027 |  |  |  |  |  |  |  |  |
| 20 | LPD49 | 0.45 | 0.55 | 0.62 | 0.38 | -2.32 | 0.028 |  |  |  |  |  |  |  |  |
| 21 | LPD25 | 0.49 | 0.51 | 0.32 | 0.68 | 2.30 | 0.029 |  |  |  |  |  |  |  |  |
| 22 | LPD352 | 0.47 | 0.53 | 0.64 | 0.36 | -2.28 | 0.030 |  |  |  |  |  |  |  |  |
| 23 | LPD190 | 0.53 | 0.47 | 0.37 | 0.63 | 2.22 | 0.035 |  |  |  |  |  |  |  |  |
| 24 | LPD77 | 0.54 | 0.46 | 0.38 | 0.62 | 2.18 | 0.038 |  |  |  |  |  |  |  |  |
| 25 | LPT140 | 0.46 | 0.54 | 0.62 | 0.38 | -2.18 | 0.038 |  |  |  |  |  |  |  |  |
| 26 | LPD79 | 0.47 | 0.53 | 0.63 | 0.37 | -2.18 | 0.038 |  |  |  |  |  |  |  |  |

**Suplementary table 2. contd…**

| **Bayes B** | | | | | | | | **Bayes C** | | | | | | | | |
| --- | --- | --- | --- | --- | --- | --- | --- | --- | --- | --- | --- | --- | --- | --- | --- | --- |
| **Sl. No.** | **Markers** | **Base Population**  **(F_2_ BP)** | | **GEBV based Selected individuals** | | **‘t’ statistic** | **‘P’ value** | **Sl. No.** | **Markers** | **Base Population**  **(F_2_ BP)** | | **GEBV based Selected individuals** | | **‘t’ statistic** | **‘P’ value** |  |
|  |  | **HA 5** | **HA 10-8** | **HA 5** | **HA 10-8** |  |  |  |  | **HA 5** | **HA 10-8** | **HA 5** | **HA 10-8** |  |  |  |
| 01 | LPT6 | 0.51 | 0.49 | 0.25 | 0.75 | 3.52 | 9.70 × 10^-4^ | 01 | LPD350 | 0.51 | 0.49 | 0.77 | 0.23 | -3.47 | 1.14 × 10^-3^ |  |
| 02 | LPD16 | 0.50 | 0.50 | 0.25 | 0.75 | 3.47 | 1.14 × 10^-3^ | 02 | LPD319 | 0.46 | 0.54 | 0.70 | 0.30 | -3.28 | 2.13 × 10^-3^ |  |
| 03 | LPT173 | 0.51 | 0.49 | 0.27 | 0.73 | 3.33 | 1.81 × 10^-3^ | 03 | LPD286 | 0.49 | 0.51 | 0.73 | 0.27 | -3.27 | 2.14 × 10^-3^ |  |
| 04 | LPD252 | 0.47 | 0.53 | 0.70 | 0.30 | -3.12 | 3.42 × 10^-3^ | 04 | LPD38 | 0.49 | 0.51 | 0.71 | 0.29 | -3.03 | 4.42 × 10^-3^ |  |
| 05 | LPD286 | 0.49 | 0.51 | 0.71 | 0.29 | -3.03 | 4.44 × 10^-3^ | 05 | LPT68 | 0.50 | 0.50 | 0.72 | 0.28 | -2.99 | 4.91 × 10^-3^ |  |
| 06 | LPD319 | 0.46 | 0.54 | 0.69 | 0.31 | -3.02 | 4.55 × 10^-3^ | 06 | LPD7 | 0.51 | 0.49 | 0.73 | 0.27 | -2.94 | 5.76 × 10^-3^ |  |
| 07 | LPD350 | 0.51 | 0.49 | 0.73 | 0.27 | -2.98 | 5.07 × 10^-3^ | 07 | LPD25 | 0.49 | 0.51 | 0.70 | 0.30 | -2.83 | 7.72 × 10^-3^ |  |
| 08 | LPD368 | 0.50 | 0.50 | 0.31 | 0.69 | 2.63 | 1.31 × 10^-2^ | 08 | LPT6 | 0.51 | 0.49 | 0.30 | 0.70 | 2.79 | 8.72 × 10^-3^ |  |
| 09 | LPT248 | 0.49 | 0.51 | 0.69 | 0.31 | -2.63 | 1.31 × 10^-2^ | 09 | LPT157 | 0.48 | 0.52 | 0.69 | 0.31 | -2.78 | 8.85 × 10^-3^ |  |
| 10 | LPD38 | 0.49 | 0.51 | 0.68 | 0.32 | -2.54 | 1.64 × 10^-2^ | 10 | LPD175 | 0.48 | 0.52 | 0.68 | 0.32 | -2.78 | 8.89 × 10^-3^ |  |
| 11 | LPT156 | 0.48 | 0.52 | 0.66 | 0.34 | -2.53 | 1.67 × 10^-2^ | 11 | LPT156 | 0.48 | 0.52 | 0.68 | 0.32 | -2.78 | 8.94 × 10^-3^ |  |
| 12 | LPT68 | 0.50 | 0.50 | 0.69 | 0.31 | -2.49 | 1.87 × 10^-2^ | 12 | LPD140 | 0.50 | 0.50 | 0.70 | 0.30 | -2.74 | 9.93 × 10^-3^ |  |
| 13 | LPD7 | 0.51 | 0.49 | 0.69 | 0.31 | -2.37 | 2.49 × 10^-2^ | 13 | LPD16 | 0.50 | 0.50 | 0.30 | 0.70 | 2.74 | 9.96 × 10^-3^ |  |
| 14 | LPT157 | 0.48 | 0.52 | 0.65 | 0.35 | -2.35 | 2.57 × 10^-2^ | 14 | LPD190 | 0.53 | 0.47 | 0.72 | 0.28 | -2.60 | 1.42 × 10^-2^ |  |
| 15 | LPD190 | 0.53 | 0.47 | 0.70 | 0.30 | -2.35 | 2.61 × 10^-2^ | 15 | LPD252 | 0.47 | 0.53 | 0.65 | 0.35 | -2.43 | 2.13 × 10^-2^ |  |
| 16 | LPD25 | 0.49 | 0.51 | 0.66 | 0.34 | -2.34 | 2.63 × 10^-2^ | 16 | LPT173 | 0.51 | 0.49 | 0.34 | 0.66 | 2.35 | 2.56 × 10^-2^ |  |
| 17 | LPD175 | 0.48 | 0.52 | 0.64 | 0.36 | -2.29 | 2.96 × 10^-2^ | 17 | LPT248 | 0.49 | 0.51 | 0.65 | 0.35 | -2.13 | 4.23 × 10^-2^ |  |
| 18 | LPD304 | 0.45 | 0.55 | 0.29 | 0.71 | 2.22 | 3.44 × 10^-2^ | 18 | LPT24 | 0.46 | 0.54 | 0.30 | 0.70 | 2.11 | 4.63 × 10^-2^ |  |
| 19 | LPD140 | 0.50 | 0.50 | 0.66 | 0.34 | -2.19 | 3.68 × 10^-2^ | 19 | LPT129 | 0.49 | 0.51 | 0.64 | 0.36 | -2.10 | 4.45 × 10^-2^ |  |
| 20 | LPT24 | 0.46 | 0.54 | 0.30 | 0.70 | 2.11 | 4.36 × 10^-2^ |  |  |  |  |  |  |  |  |  |

**Suplementary table 2. contd…**

| **Bayesian LASSO** | | | | | | | | **Bayesian Ridge Regression** | | | | | | | | |
| --- | --- | --- | --- | --- | --- | --- | --- | --- | --- | --- | --- | --- | --- | --- | --- | --- |
| **Sl. No.** | **Markers** | **Base Population**  **(F_2_ BP)** | | **GEBV based Selected individuals** | | **‘t’ statistic** | **‘p’ value** | **Sl. No.** | **Markers** | **Base Population**  **(F_2_ BP)** | | **GEBV based Selected individuals** | | **‘t’ statistic** | **‘P’ value** | |
|  |  | **HA 5** | **HA 10-8** | **HA 5** | **HA 10-8** |  |  |  |  | **HA 5** | **HA 10-8** | **HA 5** | **HA 10-8** |  |  |  |
| 01 | LPD350 | 0.51 | 0.49 | 0.76 | 0.24 | -3.27 | 1.66 × 10^-3^ | 01 | LPD350 | 0.51 | 0.49 | 0.76 | 0.24 | -3.35 | 1.66 × 10^-3^ |  |
| 02 | LPD286 | 0.49 | 0.51 | 0.73 | 0.27 | -3.25 | 2.14 × 10^-3^ | 02 | LPD286 | 0.49 | 0.51 | 0.73 | 0.27 | -3.27 | 2.14 × 10^-3^ |  |
| 03 | LPT68 | 0.50 | 0.50 | 0.74 | 0.26 | -3.02 | 2.31 × 10^-3^ | 03 | LPT68 | 0.50 | 0.50 | 0.74 | 0.26 | -3.25 | 2.31 × 10^-3^ |  |
| 04 | LPD319 | 0.46 | 0.54 | 0.69 | 0.31 | -2.99 | 4.55 × 10^-3^ | 04 | LPD319 | 0.46 | 0.54 | 0.69 | 0.31 | -3.02 | 4.55 × 10^-3^ |  |
| 05 | LPD38 | 0.49 | 0.51 | 0.71 | 0.29 | -2.94 | 4.92 × 10^-3^ | 05 | LPD38 | 0.49 | 0.51 | 0.71 | 0.29 | -2.99 | 4.92 × 10^-3^ |  |
| 06 | LPD7 | 0.51 | 0.49 | 0.73 | 0.27 | -2.83 | 5.76 × 10^-3^ | 06 | LPD7 | 0.51 | 0.49 | 0.73 | 0.27 | -2.94 | 5.76 × 10^-3^ |  |
| 07 | LPD25 | 0.49 | 0.51 | 0.70 | 0.30 | 2.79 | 7.72 × 10^-3^ | 07 | LPD25 | 0.49 | 0.51 | 0.70 | 0.30 | -2.83 | 7.72 × 10^-3^ |  |
| 08 | LPT6 | 0.51 | 0.49 | 0.30 | 0.70 | -2.78 | 8.72 × 10^-3^ | 08 | LPT6 | 0.51 | 0.49 | 0.30 | 0.70 | 2.79 | 8.72 × 10^-3^ |  |
| 09 | LPD175 | 0.48 | 0.52 | 0.68 | 0.32 | -2.78 | 8.89 × 10^-3^ | 09 | LPD175 | 0.48 | 0.52 | 0.68 | 0.32 | -2.78 | 8.89 × 10^-3^ |  |
| 10 | LPT156 | 0.48 | 0.52 | 0.68 | 0.32 | 2.74 | 8.94 × 10^-3^ | 10 | LPT156 | 0.48 | 0.52 | 0.68 | 0.32 | -2.78 | 8.94 × 10^-3^ |  |
| 11 | LPD16 | 0.50 | 0.50 | 0.30 | 0.70 | -2.66 | 9.96 × 10^-3^ | 11 | LPD16 | 0.50 | 0.50 | 0.30 | 0.70 | 2.74 | 9.96 × 10^-3^ |  |
| 12 | LPD252 | 0.47 | 0.53 | 0.67 | 0.33 | -2.62 | 1.12 × 10^-2^ | 12 | LPD252 | 0.47 | 0.53 | 0.67 | 0.33 | -2.66 | 1.12 × 10^-2^ |  |
| 13 | LPT157 | 0.48 | 0.52 | 0.67 | 0.33 | -2.60 | 1.36 × 10^-2^ | 13 | LPT157 | 0.48 | 0.52 | 0.67 | 0.33 | -2.62 | 1.36 × 10^-2^ |  |
| 14 | LPD190 | 0.53 | 0.47 | 0.72 | 0.28 | 2.59 | 1.42 × 10^-2^ | 14 | LPD190 | 0.53 | 0.47 | 0.72 | 0.28 | -2.60 | 1.42 × 10^-2^ |  |
| 15 | LPT173 | 0.51 | 0.49 | 0.32 | 0.68 | 2.46 | 1.44 × 10^-2^ | 15 | LPT173 | 0.51 | 0.49 | 0.32 | 0.68 | 2.59 | 1.44 × 10^-2^ |  |
| 16 | LPD368 | 0.50 | 0.50 | 0.32 | 0.68 | -2.30 | 1.98 × 10^-2^ | 16 | LPD368 | 0.50 | 0.50 | 0.32 | 0.68 | 2.46 | 1.98 × 10^-2^ |  |
| 17 | LPT248 | 0.49 | 0.51 | 0.66 | 0.34 | -2.29 | 2.91 × 10^-2^ | 17 | LPT248 | 0.49 | 0.51 | 0.66 | 0.34 | -2.30 | 2.91 × 10^-2^ |  |
| 18 | LPD18 | 0.52 | 0.48 | 0.69 | 0.31 | -1.95 | 2.98 × 10^-2^ | 18 | LPD18 | 0.52 | 0.48 | 0.69 | 0.31 | -2.29 | 2.98 × 10^-2^ |  |

**Suplementary table 2. contd…**

| **Random Forest Regression** | | | | | | | | **Support Vector Machine** | | | | | | | | |
| --- | --- | --- | --- | --- | --- | --- | --- | --- | --- | --- | --- | --- | --- | --- | --- | --- |
| **Sl. No.** | **Markers** | **Base Population**  **(F_2_ BP)** | | **GEBV based Selected individuals** | | **‘t’ statistic** | **‘P’ value** | **Sl. No.** | **Markers** | **Base Population**  **(F_2_ BP)** | | **GEBV based Selected individuals** | | **‘t’ statistic** | **‘P’ value** |  |
|  |  | **HA 5** | **HA 10-8** | **HA 5** | **HA 10-8** |  |  |  |  | **HA 5** | **HA 10-8** | **HA 5** | **HA 10-8** |  |  |  |
| 01 | LPT257 | 0.43 | 0.57 | 0.77 | 0.23 | -4.65 | 1.41 × 10^-5^ | 01 | LPT257 | 0.43 | 0.57 | 0.76 | 0.24 | -4.54 | 2.28 × 10^-5^ |  |
| 02 | LPD136 | 0.45 | 0.55 | 0.73 | 0.27 | -3.83 | 3.37 × 10^-4^ | 02 | LPT136 | 0.49 | 0.51 | 0.76 | 0.24 | -3.74 | 4.56 × 10^-4^ |  |
| 03 | LPD49 | 0.45 | 0.55 | 0.72 | 0.28 | -3.78 | 3.98 × 10^-4^ | 03 | LPD37 | 0.49 | 0.51 | 0.76 | 0.24 | -3.65 | 6.28 × 10^-4^ |  |
| 04 | LPT181 | 0.45 | 0.55 | 0.71 | 0.29 | -3.68 | 5.70 × 10^-4^ | 04 | LPD10 | 0.48 | 0.52 | 0.73 | 0.27 | -3.47 | 1.13 × 10^-3^ |  |
| 05 | LPD190 | 0.53 | 0.47 | 0.28 | 0.72 | 3.49 | 1.09 × 10^-3^ | 05 | LPD49 | 0.45 | 0.55 | 0.69 | 0.31 | -3.27 | 2.13 × 10^-3^ |  |
| 06 | LPT119 | 0.41 | 0.59 | 0.67 | 0.33 | -3.48 | 1.11 × 10^-3^ | 06 | LPD5 | 0.48 | 0.52 | 0.70 | 0.30 | -3.13 | 3.31 × 10^-3^ |  |
| 07 | LPD298 | 0.45 | 0.55 | 0.69 | 0.31 | -3.37 | 1.58 × 10^-3^ | 07 | LPT109 | 0.48 | 0.52 | 0.70 | 0.30 | -3.04 | 4.34 × 10^-3^ |  |
| 08 | LPT192 | 0.44 | 0.56 | 0.66 | 0.34 | -3.02 | 4.53 × 10^-3^ | 08 | LPD201 | 0.49 | 0.51 | 0.70 | 0.30 | -2.99 | 4.98 × 10^-3^ |  |
| 09 | LPT136 | 0.49 | 0.51 | 0.70 | 0.30 | -2.88 | 6.73 × 10^-3^ | 09 | LPD360 | 0.52 | 0.48 | 0.72 | 0.28 | -2.75 | 9.66 × 10^-3^ |  |
| 10 | LPD157 | 0.44 | 0.56 | 0.64 | 0.36 | -2.82 | 8.03 × 10^-3^ | 10 | LPD350 | 0.51 | 0.49 | 0.33 | 0.67 | 2.48 | 1.91 × 10^-2^ |  |
| 11 | LPD37 | 0.49 | 0.51 | 0.70 | 0.30 | -2.79 | 8.67 × 10^-3^ | 11 | LPD18 | 0.52 | 0.48 | 0.35 | 0.65 | 2.35 | 2.56 × 10^-2^ |  |
| 12 | LPD175 | 0.48 | 0.52 | 0.29 | 0.71 | 2.60 | 1.42 × 10^-2^ | 12 | LPD243 | 0.54 | 0.46 | 0.38 | 0.63 | 2.30 | 2.88 × 10^-2^ |  |
| 13 | LPD25 | 0.49 | 0.51 | 0.30 | 0.70 | 2.55 | 1.61 × 10^-2^ | 13 | LPT146 | 0.52 | 0.48 | 0.69 | 0.31 | -2.29 | 2.99 × 10^-2^ |  |
| 14 | LPT249 | 0.47 | 0.53 | 0.65 | 0.35 | -2.45 | 2.06 × 10^-2^ | 14 | LPD287 | 0.51 | 0.49 | 0.68 | 0.32 | -2.25 | 3.23 × 10^-2^ |  |
| 15 | LPD286 | 0.49 | 0.51 | 0.32 | 0.68 | 2.35 | 2.59 × 10^-2^ | 15 | LPD211 | 0.51 | 0.49 | 0.67 | 0.33 | -2.21 | 3.50 × 10^-2^ |  |
| 16 | LPT157 | 0.48 | 0.52 | 0.31 | 0.69 | 2.32 | 2.76 × 10^-2^ | 16 | LPT68 | 0.50 | 0.50 | 0.35 | 0.65 | 2.16 | 3.97 × 10^-2^ |  |
| 17 | LPD18 | 0.52 | 0.48 | 0.35 | 0.65 | 2.28 | 3.05 × 10^-2^ |  |  |  |  |  |  |  |  |  |
| 18 | LPT298 | 0.45 | 0.55 | 0.62 | 0.38 | -2.25 | 3.25 × 10^-2^ |  |  |  |  |  |  |  |  |  |
| 19 | LPD350 | 0.51 | 0.49 | 0.35 | 0.65 | 2.22 | 3.42 × 10^-2^ |  |  |  |  |  |  |  |  |  |
| 20 | LPT129 | 0.49 | 0.51 | 0.33 | 0.67 | 2.14 | 4.12 × 10^-2^ |  |  |  |  |  |  |  |  |  |
| 21 | LPT140 | 0.46 | 0.54 | 0.62 | 0.38 | -2.12 | 4.31 × 10^-2^ |  |  |  |  |  |  |  |  |  |
| 22 | LPT156 | 0.48 | 0.52 | 0.32 | 0.68 | 2.11 | 4.35 × 10^-2^ |  |  |  |  |  |  |  |  |  |
| 23 | LPT68 | 0.50 | 0.50 | 0.35 | 0.65 | 2.08 | 4.67 × 10^-2^ |  |  |  |  |  |  |  |  |  |

**Suplementary table 2. contd…**

| **Reduced Kernel Hilbert Space** | | | | | | | | **Sparse Partial Least Square** | | | | | | | | |
| --- | --- | --- | --- | --- | --- | --- | --- | --- | --- | --- | --- | --- | --- | --- | --- | --- |
| **Sl. No.** | **Markers** | **Base Population**  **(F_2_ BP)** | | **GEBV based Selected individuals** | | **‘t’ statistic** | **‘P’ value** | **Sl. No.** | **Markers** | **Base Population**  **(F_2_ BP)** | | **GEBV based Selected individuals** | | **‘t’ statistic** | **‘P’ value** |  |
|  |  | **HA 5** | **HA 10-8** | **HA 5** | **HA 10-8** |  |  |  |  | **HA 5** | **HA 10-8** | **HA 5** | **HA 10-8** |  |  |  |
| 01 | LPT173 | 0.51 | 0.49 | 0.23 | 0.77 | 3.82 | 3.54 × 10^-4^ | 01 | LPT173 | 0.51 | 0.49 | 0.27 | 0.73 | 3.28 | 1.81 × 10^-3^ |  |
| 02 | LPT6 | 0.51 | 0.49 | 0.23 | 0.77 | 3.77 | 4.22 × 10^-4^ | 02 | LPT6 | 0.51 | 0.49 | 0.27 | 0.73 | 3.23 | 2.12 × 10^-3^ |  |
| 03 | LPD16 | 0.50 | 0.50 | 0.23 | 0.77 | 3.72 | 5.02 × 10^-4^ | 03 | LPD16 | 0.50 | 0.50 | 0.27 | 0.73 | -3.22 | 2.47 × 10^-3^ |  |
| 04 | LPD286 | 0.49 | 0.51 | 0.73 | 0.27 | -3.27 | 2.14 × 10^-3^ | 04 | LPD7 | 0.51 | 0.49 | 0.75 | 0.25 | -3.03 | 2.50 × 10^-3^ |  |
| 05 | LPD350 | 0.51 | 0.49 | 0.75 | 0.25 | -3.23 | 2.46 × 10^-3^ | 05 | LPD286 | 0.49 | 0.51 | 0.71 | 0.29 | -2.98 | 4.44 × 10^-3^ |  |
| 06 | LPD38 | 0.49 | 0.51 | 0.71 | 0.29 | -3.03 | 4.42 × 10^-3^ | 06 | LPD350 | 0.51 | 0.49 | 0.73 | 0.27 | -2.79 | 5.07 × 10^-3^ |  |
| 07 | LPD319 | 0.46 | 0.54 | 0.69 | 0.31 | -3.02 | 4.55 × 10^-3^ | 07 | LPD38 | 0.49 | 0.51 | 0.70 | 0.30 | -2.77 | 8.73 × 10^-3^ |  |
| 08 | LPT68 | 0.50 | 0.50 | 0.72 | 0.28 | -2.99 | 4.91 × 10^-3^ | 08 | LPD319 | 0.46 | 0.54 | 0.67 | 0.33 | -2.64 | 9.19 × 10^-3^ |  |
| 09 | LPT156 | 0.48 | 0.52 | 0.68 | 0.32 | -2.78 | 8.94 × 10^-3^ | 09 | LPT68 | 0.50 | 0.50 | 0.70 | 0.30 | -2.54 | 1.28 × 10^-2^ |  |
| 10 | LPD368 | 0.50 | 0.50 | 0.30 | 0.70 | 2.74 | 9.93 × 10^-3^ | 10 | LPD18 | 0.52 | 0.48 | 0.70 | 0.30 | -2.53 | 1.64 × 10^-2^ |  |
| 11 | LPD7 | 0.51 | 0.49 | 0.71 | 0.29 | -2.70 | 1.11 × 10^-2^ |  |  |  |  |  |  |  |  |  |
| 12 | LPD18 | 0.52 | 0.48 | 0.71 | 0.29 | -2.69 | 1.14 × 10^-2^ |  |  |  |  |  |  |  |  |  |
| 13 | LPD25 | 0.49 | 0.51 | 0.68 | 0.32 | -2.59 | 1.46 × 10^-2^ |  |  |  |  |  |  |  |  |  |
| 14 | LPD175 | 0.48 | 0.52 | 0.66 | 0.34 | -2.54 | 1.67 × 10^-2^ |  |  |  |  |  |  |  |  |  |
| 15 | LPT157 | 0.48 | 0.52 | 0.66 | 0.34 | -2.44 | 2.11 × 10^-2^ |  |  |  |  |  |  |  |  |  |
| 16 | LPD252 | 0.47 | 0.53 | 0.65 | 0.35 | -2.43 | 2.13 × 10^-2^ |  |  |  |  |  |  |  |  |  |
| 17 | LPD140 | 0.50 | 0.50 | 0.65 | 0.35 | -2.11 | 4.34 × 10^-2^ |  |  |  |  |  |  |  |  |  |
| 18 | LPD190 | 0.53 | 0.47 | 0.69 | 0.31 | -2.09 | 4.53 × 10^-2^ |  |  |  |  |  |  |  |  |  |

**Suplementary table 2. contd…**

| **LASSO** | | | | | | | | **Phenotype-based selection** | | | | | | | | |
| --- | --- | --- | --- | --- | --- | --- | --- | --- | --- | --- | --- | --- | --- | --- | --- | --- |
| **Sl. No.** | **Markers** | **Base Population**  **(F_2_ BP)** | | **GEBV based Selected individuals** | | **‘t’ statistic** | **‘P’ value** | **Sl. No.** | **Markers** | **Base Population**  **(F_2_ BP)** | | **GEBV based Selected individuals** | | **‘t’ statistic** | **‘P’ value** |  |
|  |  | **HA 5** | **HA 10-8** | **HA 5** | **HA 10-8** |  |  |  |  | **HA 5** | **HA 10-8** | **HA 5** | **HA 10-8** |  |  |  |
| 01 | LPT6 | 0.51 | 0.49 | 0.21 | 0.79 | 4.01 | 1.75 × 10^-4^ | 01 | LPT248 | 0.49 | 0.51 | 0.34 | 0.66 | 2.10 | 4.42 × 10^-2^ |  |
| 02 | LPD16 | 0.50 | 0.50 | 0.21 | 0.79 | 3.96 | 2.11 × 10^-4^ | 02 | LPT291 | 0.51 | 0.49 | 0.36 | 0.64 | 2.09 | 4.46 × 10^-2^ |  |
| 03 | LPT173 | 0.51 | 0.49 | 0.23 | 0.77 | 3.82 | 3.54 × 10^-4^ |  |  |  |  |  |  |  |  |  |
| 04 | LPD286 | 0.49 | 0.51 | 0.71 | 0.29 | -3.03 | 4.44 × 10^-3^ |  |  |  |  |  |  |  |  |  |
| 05 | LPD252 | 0.47 | 0.53 | 0.69 | 0.31 | -2.99 | 4.99 × 10^-3^ |  |  |  |  |  |  |  |  |  |
| 06 | LPD368 | 0.50 | 0.50 | 0.29 | 0.71 | 2.90 | 6.47 × 10^-3^ |  |  |  |  |  |  |  |  |  |
| 07 | LPT68 | 0.50 | 0.50 | 0.71 | 0.29 | -2.85 | 7.40 × 10^-3^ |  |  |  |  |  |  |  |  |  |
| 08 | LPD350 | 0.51 | 0.49 | 0.72 | 0.28 | -2.85 | 7.41 × 10^-3^ |  |  |  |  |  |  |  |  |  |
| 09 | LPD7 | 0.51 | 0.49 | 0.72 | 0.28 | -2.81 | 8.15 × 10^-3^ |  |  |  |  |  |  |  |  |  |
| 10 | LPT156 | 0.48 | 0.52 | 0.68 | 0.32 | -2.78 | 8.94 × 10^-3^ |  |  |  |  |  |  |  |  |  |
| 11 | LPD25 | 0.49 | 0.51 | 0.68 | 0.32 | -2.59 | 1.46 × 10^-2^ |  |  |  |  |  |  |  |  |  |
| 12 | LPD38 | 0.49 | 0.51 | 0.68 | 0.32 | -2.54 | 1.64 × 10^-2^ |  |  |  |  |  |  |  |  |  |
| 13 | LPD175 | 0.48 | 0.52 | 0.66 | 0.34 | -2.54 | 1.66 × 10^-2^ |  |  |  |  |  |  |  |  |  |
| 14 | LPD319 | 0.46 | 0.54 | 0.65 | 0.35 | -2.51 | 1.76 × 10^-2^ |  |  |  |  |  |  |  |  |  |
| 15 | LPT157 | 0.48 | 0.52 | 0.65 | 0.35 | -2.35 | 2.57 × 10^-2^ |  |  |  |  |  |  |  |  |  |
| 16 | LPT189 | 0.48 | 0.52 | 0.64 | 0.36 | -2.20 | 3.63 × 10^-2^ |  |  |  |  |  |  |  |  |  |
| 17 | LPD190 | 0.53 | 0.47 | 0.69 | 0.31 | -2.19 | 3.69 × 10^-2^ |  |  |  |  |  |  |  |  |  |
| 18 | LPT248 | 0.49 | 0.51 | 0.65 | 0.35 | -2.13 | 4.23 × 10^-2^ |  |  |  |  |  |  |  |  |  |
